# On the role of top-down and bottom-up guidance in conjunction search: Singleton interference revisited

**DOI:** 10.3758/s13414-023-02691-8

**Published:** 2023-04-05

**Authors:** Kevin Dent

**Affiliations:** Department of Psychology, University of Essex, Wivenhoe Park, Colchester, CO4 3SQ UK

**Keywords:** Visual search, Visual attention, Conjunction search, Singleton capture, Attention capture

## Abstract

The current study reassessed the potential of salient singleton distractors to interfere in conjunction search. Experiment 1 investigated conjunctions of colour and orientation, using densely packed arrays that produced highly efficient search. The results demonstrated clear interference effects of singleton distractors in task-relevant dimensions colour and orientation, but no interference from those in a task-irrelevant dimension (motion). Goals exerted an influence in constraining this interference such that the singleton interference along one dimension was modulated by target relevance along the other task relevant dimension. Colour singleton interference was much stronger when the singleton shared the target orientation, and orientation interference was much stronger when the orientation singleton shared the target colour. Experiments 2 and 3 examined singleton-distractor interference in feature search. The results showed strong interference particularly from task-relevant dimensions but a reduced role for top-down, feature-based modulation of singleton interference, compared with conjunction search. The results are consistent with a model of conjunction search based on core elements of the guided search and dimension weighting approaches, whereby weighted dimensional feature contrast signals are combined with top-down feature guidance signals in a feature-independent map that serves to guide search.

## Introduction

The visual environment is extremely complex, and behaviourally relevant objects often appear in scenes cluttered with irrelevant objects. Selective attention refers to the set of mechanisms that allow certain stimuli to be picked out for detailed processing and the control of action, while other stimuli are disregarded (see Chun et al., 2011). Understanding the characteristics of stimuli that make them easy to find when relevant and hard to ignore when irrelevant are major goals for research in this area. Research using the visual search task has been fundamental to pursuing these research goals. In visual search, individuals make decisions about target items embedded in cluttered arrays of distractors, whilst behavioural responses are recorded. A great wealth of data has been collected regarding visual search, and this has helped improve our understanding of visual attention (see Chan & Hayward, 2013; Wolfe, 2020, for reviews).

### Efficient conjunction search and top-down control

A particularly important measure of performance in search tasks is “search efficiency,” which refers to the extent to which the time to find a target increases as additional nontargets are added to the display—essentially, the slope of the function relating RT to display numerosity. Initial experiments (Treisman & Gelade, 1980) showed that whereas targets defined by single features (e.g., red object amongst green objects) could be detected rapidly and in parallel (without much if any increase in RT for larger display sizes), objects defined by conjunctions of features (e.g., red *X* amongst red *Os* and green *X*s) were detected more slowly and with substantial increases in RT as display size increased (around 50 ms per item). Treisman and Gelade (1980) explained these observations with their feature integration theory (FIT), supposing that initial stages of visual processing recover a description of the visual scene in terms of the presence and gross distribution of a set of basic visual features including colour, orientation, size or spatial frequency, and motion, that is adequate to detect single feature but not conjunction targets. In order to detect conjunction targets each location in the display must be serially selected using spatial attention. Thus, conjunction targets should be hard to find and easy to ignore.

However, subsequent research demonstrated that conjunction search could sometimes be performed efficiently, with little cost of adding items to the display, when the features were highly discriminable. Reports of efficient search for conjunctions of colour and shape (Wolfe et al., 1989), motion and shape (McLeod et al., 1988), contrast polarity and shape (Theeuwes & Kooi, 1994), and depth and colour or motion (Nakayama & Silverman, 1986) challenged the FIT framework. In order to account for efficient conjunction search, subsequent models within the FIT family proposed that processing conjunctions of features does indeed require spatial selection but that additional mechanisms of top-down guidance are used to render this process efficient. Essentially, spatial attention may be guided towards locations likely to contain a target and away from locations unlikely to contain a target, on the basis of known target features. According to revisions of FIT (e.g., Treisman, 1988; Treisman & Sato, 1990; Wolfe, 1994; Wolfe et al., 1989), the mechanism by which this influence is exerted is a map of space coding activation at each location in the visual field. According to the guided search approach (e.g., see Wolfe, 1994, 2007, 2014, 2021) activation increases to the extent that each location contains target defining features, whereas revised FIT suggests locations containing nontarget features are instead inhibited (e.g., Treisman, 1988, 2006; Treisman & Sato, 1990; see Dent et al., 2012, for discussion). Either way, in a conjunction search for a red *X* target amongst red *Os* and green *X*s, locations containing red and locations containing *X*s end up more highly activated than locations with distractors, and spatial attention is guided towards the target location early in search, driving efficient detection.

### Stimulus-driven salience in search

In addition to top-down goal-based activation and inhibition of target and distractor features many models of search incorporate an influence of bottom-up or stimulus-driven factors in determining the speed of search. One influential theoretical idea here is that early stages of the visual system compute the “salience” of each location by calculating the similarity of each location to its neighbours within each of a set of feature dimensions. These dimension specific saliency signals are then summed together and used to direct attention and eye movements (see Itti & Koch, 2000; Koch & Ullman, 1985, for explicit implementations of this idea). According to the guided search model (e.g., Wolfe, 1994, 2021), top-down and bottom-up influences are weighted and combined to determine the overall activation of each location.

A range of evidence supports the idea that such “saliency from feature contrast” signals play an important role in search for a single feature target. One important source of evidence comes from studies investigating the influence of task irrelevant but highly salient distractors in search tasks. Theeuwes (1991, 1992) showed that the ability to select a shape-defined target (e.g., triangle amongst circles) in order to make a fine-grained decision about an embedded line was slowed down in the presence of a salient colour-defined distractor (e.g., red amongst green). One interpretation of this finding is that the rapid localization and selection of items in the display is driven by saliency signals that are feature independent, and do not differentiate relevant from irrelevant sources of saliency. As a result, participants select the most salient object on each trial, and this ends up frequently being the distractor.

Several factors modulate the impact of a salient but irrelevant distractor on search. One of these is the nature of the stimuli in the primary search task the participant is performing. Bacon and Egeth (1994) showed that if the target is not reliably a singleton in the form dimension (by including multiple targets, or by including differently shaped distractors on some trials), then an irrelevant colour distractor no longer interfered. Thus, whether participants complete a search task by selecting singletons regardless of the dimension in which they are defined may be under strategic control. According to Bacon and Egeth (1994) participants use a “singleton-detection mode” only when the target is reliably a singleton, and prefer to use a “feature search mode” in which they monitor for the presence of a particular feature when the target is not reliably a singleton. That observers may set their search goals in terms of a specific feature, or more generally in terms of any singleton, is compatible with the broader contingent capture framework (e.g., Folk & Remington, 1998; Folk et al., 1992), which suggests that stimuli will capture attention to the extent that they are task relevant. This goal could specify any salient object, any singleton, or a specific feature as task relevant, and depending on how this goal is set, different items would capture attention. Another interpretation of the Bacon and Egeth (1994) results is that participants may vary the extent to which they base selection on bottom-up saliency depending on the relative salience of the target and singleton distractor. Thus Theeuwes (2004) showed that even when the target was not reliably a shape singleton, interference from a colour distractor could be reinstated if the salience of the target and the distractor were increased by increasing display density. Tasks in which the target is difficult to find and is low salience—for example, a target defined by a relatively subtle shape difference (e.g., search for a specific letter amongst letter distractors)—may also encourage search strategies that minimize the use of salience signals such that interference is minimized (unless the distractor has specific dynamic properties like sudden onset or looming motion; see Franconeri & Simons, 2003; Jonides & Yantis, 1988). Thus, some search tasks appear to have a greater contribution from bottom-up salience signals than others, and whether these signals are used to detect targets may be at least partially under top-down control. Theeuwes (2004; see also Theeuwes, 2010, for further discussion) suggests that target salience determines the size of the attentional window that is deployed across the display—low-salience targets require a narrow attentional window that is able to exclude distractors, whereas high-salience targets encourage a wide attentional window that encompasses distractors. Similarly, other authors (e.g., Liesefeld et al., 2021a) argue that under conditions of low target salience, observers employ a strategy of systematic serial scanning of “clumps” of items in the display, rendering them relatively immune to interference from singleton distractors. In keeping with the contingent capture framework, models like guided search can accommodate such data by allowing flexible weighting of bottom-up and top-down contributions to overall activation, depending on which source of information is most useful.

Another factor modulating attentional capture is the nature of the response made in the primary search task. In contrast to the findings of Theeuwes (1992), Kumada (1999) showed that if participants were asked to judge whether a specific target was present or absent, rather than having to select a target in order to make a fine-grained discrimination about it, then cross-dimensional interference (e.g., from colour on orientation) was no longer observed. Kumada (1999) showed that if participants were asked to detect the presence of an orientation-defined target, orientation-defined distractors caused interference, but colour-defined distractors did not; likewise, orientation-defined distractors failed to interfere with search for a colour-defined target. Whilst cross-dimensional interference can be observed in present-absent search in some limited conditions (e.g., Zehetleitner et al., 2009), it remains much weaker than the equivalent within dimension interference. Thus, depending on the response required in a task, the influence of a salient distractor may vary. One explanation of this difference is that depending on the task responses may be based on different elements of the feature processing architecture. In the compound task used by Theeuwes (1992), the target must be localized, and localization requires access to a global map of space coding overall salience or activation. In the present absent task, participants may base their response on the outputs of dimension-specific feature processing modules by monitoring for any odd value in a particular dimension. Such dimensional modules are able to code feature discontinuity but do not allow the location of the discontinuity to be accessed (see also Chan & Hayward, 2009). That cross-dimensional interference can be substantially reduced or eliminated in feature search tasks that use a present–absent response is consistent with the dimensional weighting framework proposed by Müller and colleagues (e.g., Müller et al., 1995, 2003), which suggests that in feature search there are limits to the specificity with which top-down control can be exerted. According to dimensional weighting, participants may apply different weights to salience signals originating in different dimensions, such that singletons in a different dimension may be excluded, but singletons in the same dimension would continue to interfere, even when task irrelevant. Thus, whether a particular task is susceptible to interference from a task-irrelevant distractor and how that interference varies as a function of the dimension in which the distractor is defined can provide important information about how a particular search task is carried out.

### Stimulus-driven factors in conjunction search

An alternative to the idea that efficient conjunction search emerges due to top-down, feature-based activation and inhibition at the level of an activation map comes in the form of what is sometimes called “second-order parallel processing.” Beginning with the findings of efficient search for conjunctions of motion and shape (McLeod et al., 1988), various authors have suggested that the feature processing along one dimension, including bottom-up feature processing operations, may be restricted by segmentation and grouping processes to operate over particular subsets of elements. This is quite naturally illustrated in displays of moving and stationary elements, where an observer is asked to determine the presence of an *X* amongst moving *O*s and stationary *X*s. McLeod et al. suggest that under these conditions the moving items are represented completely separately to the static items by a motion filter instantiated in motion-specific processing regions of the visual cortex, and thus that form-based processing presumably including local differencing operations may be restricted to these moving items. Once segmented in this way the moving items constitute a feature search and bottom-up stimulus driven processing of this set will reveal the target.

Extending this strategy more generally Friedman-Hill and Wolfe (1995) refer this general idea as “second order parallel processing”. Second-order parallel processing in the tasks implemented by Friedman-Hill and Wolfe (1995) refers to the selection of a feature-based group of objects and then restricting the computation of dimensional difference signals (salience from feature contrast) to just the selected objects. In their subset search-task, participants knew the colour but not the orientation of the target in advance. Thus, participants might search for an oddly oriented red bar in a display of red and green bars (where the green bars shared the target orientation). The results revealed that whilst such “subset search” was possible and could show essentially parallel performance, it was much slower than typical conjunction search.

A few studies (Weidner & Müller, 2009, 2013; Weidner et al., 2002) have also examined “singleton conjunction search,” a task related to the subset search task used by Friedman-Hill and Wolfe (1995). Here participants select a subset of items on the basis of a feature in a primary dimension (e.g., all large items in a display containing large and small items, which vary in colour and motion). The defining feature of the target amongst the large items is then unpredictable—it could be unique in either colour or motion direction. Again, similarly to Friedman-Hill and Wolfe (1995), overall RTs are substantially longer than is typically observed for conjunction search, but search slopes remain flat. According to these accounts, the selected elements may behave as if they are the only elements present, and thus after an initial selection process, conjunction search becomes equivalent to feature search, and thus may suffer interference from a salient singleton, equivalent to that observed in feature search, but only when the singleton has a target feature (see also Grossberg et al., 1994; Huang & Pashler, 2007, for additional models that involve iterative segmentation processes).

Another possibility is that conjunctive differences amongst stimuli are directly represented as stimuli are treated holistically (Duncan & Humphreys, 1989; Found, 1998; Mordkoff et al., 1990; Takeda et al., 2007). If observers have access to a conjunctive salience signal, then other salient distractors may interfere with performance regardless of their properties (see Proulx, 2007, for articulation of this view).

### Is conjunction search susceptible to interference from a salient singleton distractor?

One important but unresolved issue in the literature regards the susceptibility of targets defined by conjunctions of features to singleton interference. Most of the previous work investigating the influence of a salient but irrelevant singleton on search has investigated search tasks using targets defined by single features. In conjunction search there is no one feature individuating the target, but there are two clear sources of feature-based guidance. How do these multiple influences interact? The influence of a salient but irrelevant singleton in conjunction search has the potential to be very revealing about the interplay between goal-driven and stimulus-driven influences on search. In particular, the influence of a salient singleton has the potential to reveal the extent to which conjunction search is like feature search and is subject to bottom-up stimulus-driven influences, and the extent to which it is driven by specific feature-based goals. Unfortunately, this is a question that has received relatively little study, and the studies which have addressed this issue have generated conflicting results. Lamy and Tsal (1999) asked participants to detect the presence of a green *O* target amongst red *O*s and green *T*s. On 50% of trials, a salient singleton (either a blue *O*, green *X*, or blue *X*) was present. When the target was present, there was no influence of the distractor. However, when the target was absent substantial interference was observed. The authors attributed this target-absent interference to “postselective” factors and concluded that conjunction search is driven *completely* by task goals with *no* influence from bottom-up factors. As noted by the authors situating conjunction search at this extreme upper end of the bottom-up to top-down continuum has important theoretical implications for models of search, suggesting that there is no bottom-up contribution to conjunction search, and presenting conjunction search as a paradigmatic example of top-down control of attention.

A study by Proulx (2007) also investigated the influence of a salient but completely task-irrelevant size singleton in a conjunction search. Importantly, he allowed the salient singleton to coincide with the target on some trials. Under these conditions, performance was faster when the singleton coincided with the target than when it coincided with a distractor. However, the results showed that when the singleton coincided with a distractor, it made no difference which subset of distractors it coincided with. Proulx suggested that there is a general contribution of bottom-up salience to conjunction search, but this contribution is not influenced by which goal-relevant features the singleton may happen to possess.

There are a number of differences in procedure between the studies of Lamy and Tsal (1999) and Proulx (2007) that can explain the discrepancy. Firstly, the two studies differ in terms of the relationship between the target and the irrelevant distractor. Lamy and Tsal implemented an additional singleton procedure following Theeuwes (1991, 1992), such that the salient singleton was present on half of trials and absent on the other half; when the target was present, it *never* coincided with the target. In contrast, Proulx (2007) implemented an irrelevant singleton procedure following Yantis and Egeth, (1999). Here, the singleton appears with chance probability at each stimulus location such that on target-present trials, the singleton coincides with the target on 1/*n* trials, where *n* is the display size. Proulx states that the rationale for this methodological choice was to discourage participants from strategically disabling any bottom-up contribution to search (see Gaspelin & Luck, 2018; Müller et al., 2009). If the target sometimes corresponds with the salient distractor, suppressing the salient distractor would be a suboptimal strategy since it would sometimes lead to target suppression. Indeed, previous studies have shown that when equivalent stimuli are used (e.g., Hodsoll et al., 2009) singleton interference effects can be substantially larger when the salient item sometimes coincides with the target, compared with situations where it does not. Thus, while the report of Proulx (2007) serves to show there are some circumstances under which interference with conjunction search is possible, it does not establish this as an obligatory influence that strongly runs counter to a participant’s goals.

A second difference between the two studies is the nature and salience of the singleton used. The main experiment reported by Proulx used a size singleton, an item that was larger than all the other items. This size singleton was from a different feature dimension to the task-relevant dimensions (orientation and colour) and was the only item that was different in size amongst a set of distractors that were homogeneous in size. Size provides a particularly salient feature in the context of search, something that is borne out by previous research (e.g., Proulx, 2010; Proulx & Egeth, 2008). Even in an inefficient search task where colour singletons are typically ineffective size continues to influence performance. In addition, Proulx (2007) showed that whereas a size singleton influenced a colour–orientation conjunction search, colour did not influence a size–orientation search, suggesting that size singletons can be more salient than colour singletons. Lamy and Tsal (1999) used shape and colour singletons, and previous research has demonstrated that singletons defined by shape differences often generate weak or null interference, especially when pitted against colour cues (see Theeuwes, 1991, 1992). Whilst colour singletons usually cause interference in the context of search for a shape singleton, colour singletons tend not to cause interference when the target is a letter defined by relatively subtle differences in the configuration of its component parts (e.g., Jonides & Yantis, 1988), possibly because such stimuli encourage the use of systematically serial search strategies.

Thirdly, the size singleton used by Proulx presented amongst a background of items that were homogeneous in size. Being the only item that is different in size in a size-homogeneous background would mean that the salience of the size singleton should be particularly high. In contrast, in the study by Lamy and Tsal (1999), the singleton was either unique in shape, colour, or both shape and colour, in the context of a shape–colour conjunction search. Thus, the singletons used by Lamy and Tsal were presented in a heterogenous context of distractors that had different values within the same dimension as the singleton. For example, the irrelevant singleton could be a blue item presented amongst a mixture of red and green items, this heterogeneity would likely reduce the bottom-up salience of the singleton. It is known that when a colour defined target is presented amongst a colour heterogenous background it is found less efficiently than when it appears in a homogeneous background consistent with reduced salience (see Bundesen & Pedersen, 1983; Treisman, 1988).

Thus, the study by Proulx (2007) is important as an existence proof that a sufficiently salient singleton presented in a dimensionally homogenous context can interfere with conjunction search if it sometimes coincides with the target. However, this falls short of demonstrating that such interference is automatic in the sense of being obligatory even in the face of a strong incentive to ignore the distractor. Likewise, the study by Lamy and Tsal (1999) shows that there may be some circumstances under which conjunction search can be immune to singleton interference, but this does not rule out contributions from bottom-up factors in other conjunctive settings.

Perhaps the most important feature of performance in both studies is that the search behaviour observed was not highly efficient; instead, both sets of authors found a substantial influence of display size on RT with target-present search slopes greater than 17 ms per item in both studies. This is problematic since efficient conjunction search where feature-based guidance processes are thought to play an important role typically have shallower search slopes, < 10 ms per item. This is particularly important for assessing the interaction between goal- and stimulus-driven factors, since it is not clear to what extent participants were recruiting goal-driven feature-based guidance processes *at all* in these studies. If participants did not effectively recruit feature-based guidance processes, this raises the possibility that they were engaging in a systematically serial search, perhaps with a narrowly tuned attentional window (e.g., Theeuwes, 2004). Such a serial search could take the form of the selection of one or a small number of spatially contiguous items (a “clump”) for matching against a template description of the target (see Liesefeld & Müller, 2020; Wolfe, 2021, for possible serial mechanisms). It is thus an open possibility that truly efficient conjunction search will be much more susceptible to interference from salient singleton distractors, and that under these conditions colour, and shape singletons may cause measurable interference.

One issue here is that both previous studies used relatively sparse displays of no more than 10 items, where the contribution of bottom-up salience is likely to be reduced (Nothdurft, 2000; Rangelov et al., 2017; Theeuwes, 2004). Theeuwes (2004) demonstrated that when shape targets were presented in sparse displays with heterogeneous shapes, there was no singleton interference (replicating Bacon & Egeth, 1994), but interference was reinstated with dense displays (see also Wang & Theeuwes, 2020, for a further demonstration of the importance of display density for singleton capture). Thus, display density will likely be a major factor in whether singleton interference is observed in conjunction search, as increasing display density should increase the salience of a singleton distractor. The relative salience of the target and distractor is critically important in the observation of singleton interference (e.g., see Barras & Kerzel, 2017; Theeuwes, 1992). In denser displays typical of those used to investigate conjunction search, both the target and the singleton distractor may increase in salience, but this is likely to be more pronounced for the unique singleton distractor, and more efficient search here may come at the cost of a greater distraction from salient singletons.

If a sufficiently salient singleton is introduced into an efficient conjunction search, what pattern of results should we expect? To the extent that top-down signals can be upweighted and bottom-up signals down-weighted, the influence of a salient singleton could be minimized, such that either null (e.g., Lamy & Tsal, 1999) or small effects will be found. To the extent that general dimension-independent salience continues to play a role some general effect of a salient singleton may be expected (e.g., Proulx, 2007). However, other possible patterns could emerge if there is much stronger guidance on the basis of one feature or the other. Whilst the guided search model is typically implemented with simultaneous top-down guidance from two features (e.g., Wolfe et al., 1989), it can also be implemented to allow much stronger or exclusive guidance by just one feature. If participants do indeed operate by using one primary feature to guide search, then we should expect to find stronger interference from a salient distractor when it shares the dominant target feature. Thus, if participants use colour to guide search, then orientation singletons in the target colour should interfere more than singletons without this colour. Likewise, if orientation is used to guide search, colour singletons in the target orientation should interfere more than colour singletons in the nontarget orientation. Thus, observing which target features modulate distractor interference in conjunction search will provide important information about which strategies participants use to guide search for conjunction targets (see also Proulx, 2007).

### The current study: Reassessing singleton capture in conjunction search

The aim of the current study was to address the issues raised above with the studies of Lamy and Tsal (1999) and Proulx (2007) and to reassess the interference from a salient singleton in conjunction search. The current experiments sought to explore several questions. The first question concerned whether singleton capture could be observed in conjunction search at all under conditions where the singleton and target never coincide (unlike Proulx, 2007). Demonstrating interference from salient singletons would support an important role for bottom-up salience signals in conjunction search and seriously undermine the claim (e.g., Lamy & Tsal, 1999) that conjunction search is under complete control of feature-based goals.

The second question concerned whether any interference would be impacted by the relevance of the dimension in which the singleton was defined. According to the dimension weighting model (e.g., Müller et al., 2003; see Liesefeld et al., 2018; Liesefeld et al., 2019, for reviews), participants are able to control the specific feature dimensions that are most heavily weighted in the calculation of salience. In order to address this question in the context of search for conjunctions of orientation and colour, singletons defined in the domain of motion (moving elements) known to be salient but defined in a task-irrelevant dimension were included.

The third question concerned whether singleton interference would be modulated by target features. In order to address this question when the singleton was defined in a task-relevant dimension, its feature on the other task-relevant dimension was systematically varied. Thus, a colour singleton either appeared with target or nontarget orientation, and an orientation singleton either appeared with a target or nontarget colour. If bottom-up signals are impervious to top-down control—for example, if a salience map codes only bottom-up salience in a fixed an inflexible fashion (e.g., the salience map of Theeuwes, 2010)—then singletons should interfere regardless of whether they possess target features. In contrast, if an activation map combines both top-down and bottom-up influences, target features should modulate singleton interference, and the exact pattern of modulation will provide evidence of which features are used to guide search (colour, orientation, or both). Relatedly, it was of interest whether one target-relevant feature would dominate the other—in the case of colour and orientation, will interference from a singleton show a greater degree of modulation by sharing colour or by sharing orientation with the target?

The final question concerned the qualitative pattern of singleton interference in feature compared with conjunction search. To address this question, the conjunction search task used in Experiment 1 was reconfigured in Experiments 2 and 3 to provide a feature search baseline. The relative susceptibility of conjunction and feature search to singleton interference provides theoretically important information regarding whether these two types of visual search task are fundamentally similar and rely on a shared set of psychological mechanisms or whether conjunction search is special. Observing singleton interference in both tasks would argue in favour of a continuous set of mechanisms operating in both tasks.

## Experiment 1

In Experiment 1, participants were asked to complete a standard conjunction search for a target defined by colour and orientation (e.g., Wolfe et al., 1989). Colour–orientation conjunction search was implemented since previous research has established that this is a task that can be performed highly efficiently (see Friedman-Hill & Wolfe, 1995; Wolfe et al., 1989). For half of the participants, the target was a vertically oriented red bar amongst vertically oriented green bars and horizontally oriented red bars. For the other half of the participants, the colour assignments were reversed such that participants searched for a green vertical bar amongst green horizontal bars and red vertical bars. The task was simply to indicate whether the target was present (50% of trials) or absent (50% of trials). Regardless of target presence, a singleton distractor was present on 50% of trials. This singleton distractor was defined either in the orientation dimension (a 45° tilted bar), in the colour dimension (a blue bar), or in the motion dimension (a moving bar, oscillating left and right). Orientation was used rather than more complex shapes since recent research has demonstrated strong within-dimension interference in the orientation domain (Kumada, 1999; Liesefeld et al., 2017, 2019; Sauter et al., 2018). For example, Kumada (1999) reported within-dimension orientation interference of some 200 ms. Given that interference effects with orientation are known to be robust, using orientation as one of the task-relevant dimensions will provide a sensitive test of singleton interference in conjunction search.

When present, the salient singleton replaced either one of the two types of conjunction distractor equally often. When the singleton replaced one of the distractors, the nonsingleton properties were maintained. Thus, the orientation singleton was equally often red or green, the colour singleton was equally often vertical or horizontal, and the moving distractor was equally often a green vertical or red horizontal item (for participants with a red vertical target).

Different patterns of interference are to be expected, depending on how participants approach this task and depending on the mechanisms by which top-down and bottom-up cues interact in conjunction search. If participants exert precise feature-based control, and select targets primarily on the basis of top-down feature activations very little interference from irrelevant singletons should be expected (e.g., Bacon & Egeth, 1994; Lamy & Tsal, 1999). To the extent that participants are generally sensitive to salience from feature contrast (e.g., Proulx, 2007; Theeuwes, 2010), they may be generally disrupted by the presence of singletons, but they should be most distracted by the motion singleton since this item is the most distinctive, differing in motion from all other items, whereas orientation and colour singletons occur in the context of other orientation and colour heterogeneities (and within-dimension heterogeneity is known to influence salience; e.g., Treisman, 1988).

The type of oscillating motion used was similar to that used in earlier studies by Treisman and Sato (1990), Hillstrom and Yantis (1994), and Yantis and Egeth (1999). These studies establish that oscillating motion behaves much like colour in the sense that it provides a salient featural discontinuity that can be used to rapidly and efficiently detect a target, but that when task irrelevant, it does not necessarily lead to interference, at least when search is inefficient. To the extent that participants engage in dimension-based weighting of these bottom-up salience signals (e.g., see Liesefeld et al., 2017, 2019), it should be possible to down-weight signals from the motion dimension, leaving only the colour or orientation singletons to interfere. To the extent that participants are able to use feature-based control to emphasize salience at certain locations (e.g., Friedman-Hill & Wolfe, 1995; Wolfe, 1994), we should expect that singletons with target features should interfere more than those without.

### Method

The methods used in the work described in this article were approved by the University of Essex Ethics Committee (Project KD1101).

#### Participants

Previous research using similar methods used eight (Kumada, 1999) participants, and sample sizes between eight and 20 are typical of research in this area. The current research used a sample size of 16 participants that was set in advance of beginning the research. The degree of interference typically observed in the additional singleton paradigm varies quite widely. Interference is typically larger for singletons defined in the target feature dimension and larger in compound than simple present–absent tasks. The study by Kumada (1999) is useful in order to estimate the typical effect size to be expected for within-dimension interference in a simple present–absent task similar to the one used here. Kumada reported a within-dimensional capture effect of around 110 ms in a simple search task, giving an effect size of η_p_^2^ = 0.87; this value provides a useful empirically derived baseline for the general size of the interference effect we would wish to detect. Using the effect size for the overall capture effect in Kumada’s (1999) Experiment 1, the G*Power program showed that a sample size of five participants would be required to detect this effect at a significance level of 0.05 for a power of 0.95. In order to estimate the minimal effect size that is of theoretical relevance, we took the classic study by Theeuwes (1992), where cross-dimensional interference of colour on a form feature search is around 25 ms, and gives an effect size of η_p_^2^ = 0.67, the G*Power program showed that a sample size of 10 participants would be required to detect this effect at a significance level of 0.05 for a power of 0.95. Given the way in which the data is analyzed below, the interference effect as reported by Kumada (1999) and Theeuwes (1992) corresponds to the *t* test testing whether the singleton interference is greater than zero. Sixteen participants thus should provide adequate power to detect any interference effects of theoretical relevance. Sixteen participants from the staff and students at the University of Essex volunteered in exchange for either course credit or for a payment of £5. There were five male and 11 female participants, all of whom were right-handed (mean age = 21.7, range: 18–29 years).

#### Equipment

The experiment was controlled by programs written with the Psychophysics Toolbox (Brainard, 1997; Pelli, 1997) and MATLAB 8.0. Stimuli were displayed on a Mitsubishi 2070SB 21-in. monitor (120 Hz), driven by a Mac Pro equipped with an ATI 5870 graphics card. Participants interacted with the computer via a standard keyboard.

#### Stimuli

**Fig. 1:**
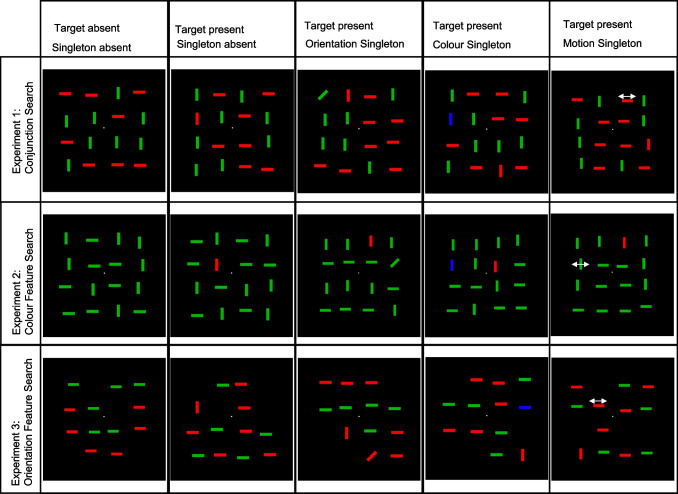
Illustration of the search displays used across all three experiments. *Note.* Motion is illustrated using a horizontal arrow. Experiments 1 and 2 are illustrated using 16 item displays, and Experiment 3 is illustrated using 12 item displays. (Colour figure online)

The displays were viewed from a distance of approximately 57 cm, although participants did not use a chin rest. The search displays were constructed from a set of bar-shaped items, each bar was 0.3 cm (0.3 degrees) in width and 1.2 cm (1.2 degrees) in length (see Fig. 1 for example displays, and Table 1 for an exhaustive list of the different targets and distractors that could occur). The basic (singleton absent) search displays were composed of vertical and horizontal bars, that were green (RGB value [0 180 0]), or red (RGB value [255 0 0]) in colour, with the two colours matched to be approximately equiluminant for the author by heterochromatic flicker photometry (nonsingleton distractors).

**Table 1:** Search items and their features used in conjunction search (Experiment 1)

Group	Dimension	Target	Nonsingleton distractors		Singleton distractor		
			Target Orientation	Target Colour	Colour	Orientation	Motion
Group 1	Colour	Red	Green	Red	Blue	Red / Green	Red / Green
	Orientation	Vertical	Vertical	Horizontal	Vert / Horiz	45°	Horiz / Vert
	Motion	0 cm/s	0 cm/s	0 cm/s	0 cm/s	0 cm/s	1.8 cm/s
Group 2	Colour	Green	Red	Green	Blue	Red / Green	Red / Green
	Orientation	Vertical	Vertical	Horizontal	Vert / Horiz	45°	Vert / Horiz
	Motion	0 cm/s	0 cm/s	0 cm/s	0 cm/s	0 cm/s	1.8 cm/s

*Note.* Vertical and horizontal are occasionally abbreviated as *vert* and *horiz* due to space constraints

For half of the participants the target was a red vertical bar, and for the other half it was a green vertical bar, such that when present, targets shared orientation but not colour with one set of nonsingleton distractors (target orientation nonsingleton distractors), and colour but not orientation with the remaining nonsingleton distractors (target colour nonsingleton distractors). In displays where neither a target nor a singleton distractor was present, the two kinds of nonsingleton distractors were present in equal numbers. The target when present replaced either one of the two distractor types equally often. When participants searched for a red vertical bar, the nonsingleton distractors were green vertical and red horizontal bars. When participants searched for a green vertical bar, the nonsingleton distractors were red vertical and green horizontal bars. For clarity, the colour and orientation features that define the target are referred to as target features, and the colour and orientation features that are always present in the display but do not define the target are nontarget features.

Each bar was positioned in the cells of a 4 × 4 matrix (modelled on that used by Friedman-Hill & Wolfe, 1995), with each cell spaced by 2.42 cm (2.42 degrees). Search items were initially assigned to the centre of a cell before being jittered by random distance up to 0.6 cm in both directions, the maximum array dimensions were thus 11.2 × 11.2 cm (11.2 × 11.2 degrees). On each trial, 12 or 16 bars were presented.

On 50% of trials, a salient but irrelevant singleton distractor replaced one of the nonsingleton distractors. The singleton distractor was defined in either the colour, orientation, or motion dimension equally often (33.33% of the singleton distractor present trials). The colour singleton distractor was always blue (RGB value: [0 0 255]), the orientation singleton distractor was always a 45° tilted bar (similar to “/” with antialiasing used to prevent jagged edges), and the motion singleton distractor always oscillated horizontally through 0.6 cm (0.6 degrees) at a speed of 1.8 cm/second (1.8 degrees/second).

Each singleton distractor replaced each type of nonsingleton distractor (target orientation or target colour) equally often, and the implications of which type of distractor was replaced differed according to the dimension in which the singleton distractor was defined. Thus, the blue colour singleton distractor was equally often vertical or horizontal, meaning that it shared the orientation of the target on 50% of occasions and shared the orientation only of some nonsingleton distractors on the remaining 50% of trials. The right-tilted orientation singleton distractor was equally often red or green, meaning that it shared the colour of the target on 50% of trials and shared the colour of some nonsingleton distractors on the remaining 50% of trials. In the case of the moving singleton distractor, this item was equally often presented with the target colour (either red or green, depending on group) but nontarget orientation (horizontal), or with the target (orientation vertical) but nontarget colour (either red or green, depending on participant group).

#### Design and procedure

At the start of the experiment, participants were given verbal instructions by the experimenter. They were instructed to search for the target item and to ignore any nontarget distractors. They were shown examples of the target-present and target-absent displays and examples of the types of salient singleton distractor displays that could appear. Participants then completed a block of 48 practice trials, during which they could ask questions of the experimenter, and during which the experimenter verified that the participants were completing the task correctly. Following this initial set of practice trials, participants were given an additional set of 48 practice trials. During the practice, accuracy was monitored online, and practice would be automatically reapplied if accuracy was less than 85%; participants were informed of this contingency. Once the practice was complete, participants completed 960 trials, broken down into six blocks of 160 trials. In between blocks of trials, participants were encouraged to take a short break. The presentation of trials paused in between blocks, and the text “Take a Break” appeared until participants pressed a key to begin the next block.

Each trial began with the presentation of a small fixation dot (0.12 cm, 0.12 degrees in diameter) in the centre of the screen for 250 ms. The search items were then added to the screen and remained present until the participant made a response. Half the participants pressed the *z* key to indicate target present and the *n* key to indicate target absent, and for the other half, the key assignment was reversed. Following a response, participants were given feedback about their accuracy on every trial. Following a correct response, participants were shown a schematic smiling face, whereas following an incorrect response, participants were shown a schematic frowning face. The face appeared for 400 ms, followed by a 100-ms blank screen. Participants were instructed to respond as quickly but as accurately as possible. All possible trial types appeared in a random order within each block. For singleton-present trials, there were 20 repetitions of each of the critical types of trial created by combining the factors: target presence (present vs. absent), singleton dimension (colour, orientation, vs. motion), display size (12 or 16 items), and the nonsingleton features of the singleton distractor. In relation to the nonsingleton features of the singleton distractor, this took a different form for each of the three types of singleton distractor. In the case of orientation-singleton distractors, the item either shared the colour of the target or was presented in the nontarget colour. In the case of the colour-singleton distractors, the item either shared the orientation of the target or was presented in the nontarget orientation. In the case of the motion-singleton distractor, the item was either presented with the target colour but nontarget orientation, or the target orientation but nontarget colour. For singleton-absent trials, there were 120 trials of each of the critical trial types given by combining: target presence (present vs. absent) and display size (12 or 16 items).

### Results

The data used in the analyses reported are publicly available online (https://osf.io/wbr2p/). Incorrect trials (4.84%) and trials with RTs greater than 2,000 ms (1.49%) were excluded from the RT analyses. Accuracy was overall very high (see Table 2A and 2B) and whilst analyses of RT are foregrounded equivalent analyses of accuracy are reported for completeness.

**Table 2:** Accuracy levels (proportion correct) in Experiment 1. A: data for singleton distractor present trials. B: data for singleton distractor absent trials

A: Accuracy levels (proportion correct) in Experiment 1 showing singleton-present trials								
	Target absent				Target present			
Colour	Target orientation		Nontarget orientation		Target orientation		Nontarget orientation	
	0.98	0.97	0.96	0.97	0.92	0.92	0.94	0.91
Orientation	Target colour		Nontarget colour		Target colour		Nontarget colour	
	0.95	0.94	0.98	0.98	0.94	0.91	0.92	0.91
Motion	Target colour		Target orientation		Target colour		Target orientation	
	0.98	0.97	0.98	0.98	0.93	0.92	0.95	0.91
Display size	12	16	12	16	12	16	12	16
B: Accuracy levels (proportion correct) in Experiment 1 showing singleton-absent trials								
Singleton absent	Target absent				Target present			
	0.97		0.98		0.94		0.93	
Display size	12		16		12		16	

#### Baseline search performance

**Fig. 2:**
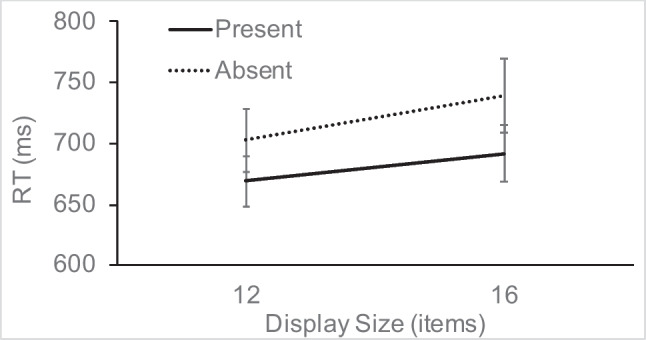
Experiment 1 search RTs for target-present and target-absent trials as a function of display size, on singleton-absent trials (error bars show standard errors)

RT in the singleton-absent trials (see Fig. 2) was analyzed using a 2 × 2 repeated-measures analysis of variance (ANOVA), with the factors target presence (present vs. absent) and display size (12 or 16). The main effect of target presence was significant, *F*(1, 15) = 25.96, η_p_^2^ = 0.63, *p* < 0.001, as was display size, *F*(1, 15) = 8.14, η_p_^2^ = 0.35, *p* = 0.012, but the interaction did not reach significance, *F*(1, 15) = 1.59, η_p_^2^ = 0.1, *p* = 0.226. RTs were slower on target-absent trials and showed an overall small increase of 9 ms/item as a function of display size.

Accuracy was analyzed with an ANOVA, with the same structure as that used to analyze RT. The main effect of target presence was significant, *F*(1, 15) = 22.87, η_p_^2^ = 0.60, *p* < 0.001, such that target-present trials were slightly less accurate than target-absent trials (94 vs. 98%); there was no effect of display size, *F* < 1, and no interaction, *F*(1, 15) = 2.70, η_p_^2^ = 0.15, *p* = 0.121.

#### Interference effects

**Fig. 3:**
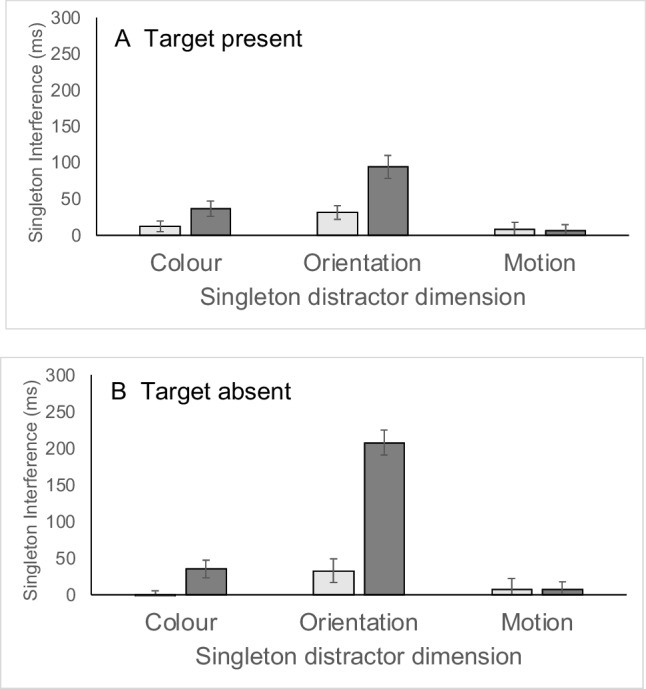
Experiment 1 singleton interference effects (singleton present − singleton absent) in milliseconds, as a function of singleton dimension (horizontal axis) and nonsingleton features. *Note.* For colour and orientation singletons, light-grey bars show interference scores for singletons that had no target features, and dark-grey bars show singleton interference for singletons that shared either orientation (colour singleton) or colour (orientation singleton) with the target. In the case of motion singletons, light-grey bars are for singleton distractors with the target orientation and nontarget colour, whereas dark-grey bars are for singletons with the target colour but nontarget orientation. Panel **A** shows data for target-present trials, whereas Panel **B** shows data for target-absent trials

Singleton interference effects were calculated as the difference in either RT or accuracy between singleton-distractor-present and singleton-distractor-absent responses (see Fig. 3 for RT costs). Interference effects for singleton distractors defined in each of the three dimensions were analyzed separately using three-factor ANOVAs, with the factors of target presence (target present vs. target absent), display size (12, 16), and nonsingleton features of the singleton distractor (the levels here depend on the specific analysis). Three statistical effects were of particular theoretical relevance. Firstly, the overall interference effect tested with a one-sample *t* test against a test value of zero ms indexed whether the disruption from the salient singleton was greater than zero, for each dimension. Secondly, the main effect of target presence in the ANOVA, indexed whether there was any difference in interference on present and absent trials. This is important since previous research has attributed particular importance to effects that are found on target-present trials (e.g., Lamy & Tsal, 1999). Thirdly, the main effect of nonsingleton features of the singleton distractor in the ANOVA indexed the extent to which singleton interference varies according to whether the singleton shares various features with the target. This is most interesting in the cases of colour and orientation singletons, since here this factor indicates whether the singleton shares a feature with the target or not. If top-down control is able to modulate bottom-up salience, then an effect of the nonsingleton feature of the singleton distractor is to be expected.

##### Colour

The RT analysis showed that the overall interference effect was significantly different from zero, *t*(15) = 3.59, Cohen’s *d* = 0.90, *p* = 0.003. An ANOVA with the factors target presence (target present vs. target absent), display size (12, or 16), and nonsingleton features of the singleton distractor (target orientation, nontarget orientation) revealed a main effect of the nonsingleton feature of the singleton distractor, *F*(1, 15) = 21.48, η_p_^2^ = 0.59, *p* < 0.001. Separate one-sample *t* tests for singletons with and without the target orientation revealed a significant overall interference effect for distractors with the target orientation, *t*(15) = 4.59, Cohen’s *d* = 1.15, *p* < 0.001, but no effect, *t*(15) = 0.98, Cohen’s *d* = 0.24, *p* = 0.35, for distractors with the nontarget orientation. There was no effect of target presence, *F*(1, 15) = 0.47, η_p_^2^ = 0.03, *p* = 0.51, and no effect of display size, *F*(1, 15) = 0.01, η_p_^2^ = 0.00,* p* = 0.932. The interactions between target presence and nonsingleton feature of the singleton distractor, *F*(1, 15) = 1.17, η_p_^2^ = 0.07, *p* = 0.296, target presence and display size, *F*(1, 15) = 0.00, η_p_^2^ = 0.00, *p* = 0.987, nonsingleton feature of the singleton distractor and display size, *F*(1, 15) = 0.53, η_p_^2^ = 0.03, *p* = 0.478, and the three-way interaction between all factors, *F*(1, 15) = 2.27, η_p_^2^ = 0.13, *p* = 0.153, were nonsignificant.

Regarding accuracy, the overall interference effect was not significant, *t*(15) = 1.98, Cohen’s *d* = 0.50, *p* = 0.066. An ANOVA with the same structure as that used to analyze RT revealed no statistically significant effects, all *F*(1, 15) < 1.51, η_p_^2^ < 0.09, *p*s > 0.230.

##### Orientation

The analysis of RT revealed that the overall interference effect was significantly different from zero, *t*(15) = 8.69, Cohen’s *d* = 2.17, *p* < 0.001. An ANOVA with the factors target presence (target present vs. target absent), display size (12, or 16) and nonsingleton features of the singleton distractor (target colour, nontarget colour) was also used to analyze the data. The main effect of target presence, *F*(1, 15) = 19.98, η_p_^2^ = 0.57, *p* < 0.001, was significant with stronger interference on absent than present trials. The main effect of the nonsingleton feature of the singleton distractor, *F*(1, 15) = 103.09, η_p_^2^ = 0.87, *p* < 0.001, was also significant, with stronger orientation interference when the singleton distractor appeared in the target colour, these two factors also interacted, *F*(1, 15) = 19.29, η_p_^2^ = 0.56, *p* < 0.001. Separate analyses for singleton distractors with and without target colour were carried out to explore the Target Presence × Nonsingleton Properties interaction. When the orientation singleton distractor appeared in the nontarget colour, interference was significantly greater than zero, *t*(15) = 4.54, Cohen’s *d* = 1.14, *p* < 0.001; however, an ANOVA revealed no effect of target presence, *F*(1, 15) = 0.00, η_p_^2^ = 0.00, *p* = 0.966, consistent with similar effects on present and absent trials. In contrast, when the singleton appeared in the target colour, there was an overall interference effect, *t*(15) = 9.75, Cohen’s *d* = 2.44, *p* < 0.001, and an ANOVA revealed that interference was greater on target-absent than on target-present trials, *F*(1, 15) = 90.98, η_p_^2^ = 0.86, *p* < 0.001. In the overall ANOVA no other effects were significant, display size, *F*(1, 15) = 1.80, η_p_^2^ = 0.11, *p* = 0.2; Target Presence × Display Size, *F*(1, 15) = 0.02, η_p_^2^ = 0.00,* p* = 0.900; Nonsingleton Properties × Display Size, *F*(1, 15) = 0.03, η_p_^2^ = 0.00, *p* = 0.866; three-way interaction, *F*(1, 15) = 0.39, η_p_^2^ = 0.03, *p* = 0.541.

The overall interference effect on accuracy was not significant, *t*(15) = 2.12, Cohen’s *d* = 0.53, *p* = 0.051. However, an ANOVA, with the same structure as that used to analyze RT, revealed the interaction between target presence and the nonsingleton properties of the singleton distractor was significant, *F*(1,b15) = 5.37, η_p_^2^ = 0.26, *p* = 0.035. Separate analyses of target-present and target-absent trials showed that for target-present trials, there was no significant overall interference, *t*(15) = 1.62, Cohen’s *d* = 0.41, *p* = 0.125, nor did the ANOVA reveal any effects of the nonsingleton properties of the singleton distractor, *F* < 1. In contrast for target-absent trials, the overall interference effect was significant, *t*(15) = 2.14, Cohen’s *d* = 0.54, *p* = 0.049, and an ANOVA revealed that there was an effect of the singleton properties of the singleton distractor, *F*(1, 15) = 8.27, η_p_^2^ = 0.36, *p* = 0.012. Only when the singleton distractor shared the colour of the target was there significant interference, *t*(15) = 2.61, Cohen’s *d* = 0.65, *p* = 0.02; in contrast, when the singleton distractor did not share target colour, the interference effect was not significant, *t*(15) = 1.03, Cohen’s *d* = 0.26, *p* = 0.319. In the overall ANOVA, no other effects were significant, display size, *F*(1, 15) = 1.59, η_p_^2^ = 0.10, *p* = 0.227; Target Presence × Display Size; Nonsingleton Properties × Display Size; and the three-way interaction, *F*s < 1.

##### Motion

The analyses of RT showed that the interference effect, was not significantly different from zero, *t*(15) = 1.85, Cohen’s *d* = 0.46, *p* = 0.084, although there was a trend towards interference. An ANOVA with the factors target presence (target present vs. target absent), display size (12 or 16) and nonsingleton features of the singleton distractor (target orientation but nontarget colour, target colour but nontarget orientation) revealed no main effects or interactions; target presence, *F*(1, 15) = 0.00, η_p_^2^ = 0.00, *p* = 0.977; nonsingleton features of the singleton distractor, *F*(1, 15) = 0.01, η_p_^2^ = 0.00, *p* = 0.939; display size, *F*(1, 15) = 0.01, η_p_^2^ = 0.00, *p* = 0.923; Target Presence × Nonsingleton Features of the singleton distractor, *F*(1, 15) = 0.02, η_p_^2^ = 0.00, *p* = 0.880; Target Presence × Display Size, *F*(1, 15) = 0.49, η_p_^2^ = 0.03, *p* = 0.495; nonsingleton features of the Singleton Distractor × Display Size, *F*(1, 15) = 0.51, η_p_^2^ = 0.03, *p* = 0.488; three-way, *F*(1, 15) = 0.25, η_p_^2^ = 0.02, *p* = 0.626.

Regarding accuracy the overall interference effect was unambiguously nonsignificant, *t*(15) = 0.76, Cohen’s *d* = 0.19, *p* = 0.459. However, an ANOVA with the same structure as that used for RT revealed an interaction between all three factors that just reached significance, *F*(1, 15) = 4.62, η_p_^2^ = 0.24, *p* = 0.048. Neither when the singleton distractor shared the target orientation or when it shared target colour was the overall interference effect significant, *t*s(15) < 1.10, Cohen’s *d* < 0.26, *p* > 0.324. When the singleton distractor shared target colour ANOVA did not reveal any significant effects, *F*s(1, 15) < 2.10, η_p_^2^ < 0.12, *p* > 0.168. However, when the singleton distractor shared the target orientation the interaction between target presence and display size was significant, *F*(1, 15) = 4.62, η_p_^2^ = 0.24, *p* = 0.048. This in turn was driven by an effect of display size that was significant only for target-present, *F*(1, 15) = 7.34, η_p_^2^ = 0.33, *p* = 0.016, but not target-absent trials, *F*(1, 15) < 1. On target-present trials, when the singleton shared the target orientation, there was some small interference but only for a display size of 16, *t*(15) = 2.48, Cohen’s *d* = 0.62, *p* = 0.025, and not 12, *t*(15) = 1.14, Cohen’s *d* = 0.29, *p* = 0.271. Thus, there is a tiny interference effect of 2.7% on accuracy that is limited to present trials, for singletons that share target orientation, in Display Size 16.

##### Comparing RT interference from colour and orientation

A combined analysis of colour and orientation RT singleton-distractor interference was carried out using a four-factor ANOVA. The factors of target presence (present vs. absent) and display size (12 vs.16) were used as in the above analyses. The factor of singleton-distractor dimension was added (colour vs. orientation), and nonsingleton feature of the singleton distractor was coded simply in terms of whether the singleton distractor shared a feature with the target or not (colour in the case of the orientation singleton and orientation in the case of the colour singleton). The three-way interaction between singleton-distractor dimension, target presence, and nonsingleton feature of the singleton distractor was significant, *F*(1, 15) = 11.80, η_p_^2^ = 0.44, *p* = 0.004.

Separate analyses by nonsingleton feature of the singleton distractor showed that when the singleton did not share a feature with the target, overall the interference effect was greater than zero ms, *t*(15) = 3.85, Cohen’s *d* = 0.96, *p* = 0.002, and the ANOVA revealed an effect of singleton-distractor dimension, *F*(1, 15) = 12.56, η_p_^2^ = 0.46, *p* = 0.003, with greater interference when singletons were defined in the orientation than the colour dimension, but no interaction between target presence and singleton-distractor dimension, *F*(1, 15) = 0.73, η_p_^2^ = 0.05, *p* = 0.406. When the singleton distractor shared a feature with the target, interference was greater than zero ms, *t*(15) = 9.62, Cohen’s *d* = 2.40, *p* < 0.001, and the ANOVA revealed an effect of singleton-distractor dimension, *F*(1, 15) = 58.67, η_p_^2^ = 0.80, *p* < 0.001, an effect of target presence, *F*(1, 15) = 27.07, η_p_^2^ = 0.64, *p* < 0.001, and an interaction between target presence and singleton-distractor dimension, *F*(1, 15) = 40.74, η_p_^2^ = 0.73, *p* < 0.001, consistent with the significant effect of target presence for orientation but not colour defined singleton distractors documented above.

### Discussion

The results revealed a pattern of highly efficient search with little change in RT as a function of display size (6 ms/item for target-present trials), consistent with a pattern of parallel processing of multiple items. However, this efficient target detection was not always accompanied by efficient rejection of salient singleton distractors. Singleton distractors often caused significant interference that was largely restricted to RT having only the most restricted effect on accuracy. In particular, considering the theoretically most pertinent target-present trials, orientation and colour singleton distractors only disrupted RT, having no effect on accuracy at all. Singleton distractors from a task-relevant dimension (e.g., colour and orientation) caused much greater interference compared with singleton distractors defined by the presence of motion. Singleton distractors defined by motion caused no significant interference at all as measured by RT, even on absent trials, which in general are more susceptible to interference in the current data set. There was some very small (2.7% on accuracy) but significant interference caused by motion singletons that was limited to cases where the singleton shared orientation with the target, but only for target present trials and only for the larger display size. The results thus implicate a substantial role for top-down modulation of salience from dimensional feature contrast information in this task. Motion singletons have only an extremely limited influence on performance. Thus, participants are not operating in a way that they are primarily guided purely by global or first-order calculations of salience. Participants are able to appropriately reject salient singletons that originate in the motion dimension. On some trials, this rapid rejection of the motion singleton can sometimes lead to participants missing the target, but this effect is negligible. Thus, it appears that participants are not operating in a “singleton detection” mode (e.g., Bacon & Egeth, 1994), nor are they using an unfiltered dimension general salience signal (e.g., Theeuwes, 2004); participants are able to exclude some singleton distractors. Note that this result is in contrast to the finding of Proulx (2007) with a size singleton, and is consistent with the idea that when a cross-dimensional singleton never corresponds to the target location, it may be safely suppressed prior to being selected (see Gaspelin & Luck, 2018).

The current results demonstrate clear limits on the ability of participants to control interference, as shown by the substantial colour- and orientation-singleton-distractor interference. The finding of substantial interference from singleton distractors in task-relevant dimensions on target-present trials is clearly at odds with the results of Lamy and Tsal (1999) and contradicts the idea that conjunction search operates in Bacon and Egeth’s (1994) feature search mode. Note that the colour and orientation singleton-distractor interference on present trials was restricted to RT and occurred in the absence of interference with accuracy. Accuracy on target-absent trials was slightly worse in the presence of an orientation singleton, suggesting that participants sometimes made false-alarm errors in response to the orientation singleton. Isolating the exact reason why the current results reveal clear RT interference on target-present trials, whereas the previous results did not require further experiments. One likely contributing factor is that participants in the current experiment demonstrated highly efficient search, such efficient search makes it unlikely that participants engaged in a systematically serial search strategy such as “clump scanning” (e.g., see Liesefeld et al., 2021a; Liesefeld & Müller, 2020) or set an attentional window narrowly (e.g., Theeuwes, 2004), both of which might lead to reduced singleton interference. Increased display density in the current experiment is also likely to have served to increase the salience of the distractor, rendering interference observable (e.g., Theeuwes, 2004).

Of critical importance, the current results show that colour and orientation signals do modulate one another. It is not the case that any colour or orientation singleton impacts performance equally. Interference is very tightly modulated by task relevance on the nonsingleton dimension. Thus, colour interferes *only* when the colour singleton distractors have the target orientation, and likewise orientation interferes much more strongly when orientation singleton distractors have the target colour. Participants find it difficult to ignore items that have an odd value in a target-relevant dimension indicating that they operate to some extent on the basis of dimensional salience computed within task-relevant dimensions. However, there is then some additional feature-based mechanism that must operate to modulate these signals top down, so that signals that apply to items with task-relevant features are accentuated.

The guided search model provides a mechanism whereby feature-based guidance can influence the use of dimensional-salience signals at the level of the activation map, where top-down, feature-based signals are summed with bottom-up, dimensional contrasts. The guided search model emphasizes top-down, feature-based control and supposes that in the case of conjunction search, since bottom-up salience signals are not useful, adding only noise, generally, their contribution should be minimized. In contrast, the dimension weighting model that is typically used to explain search for targets defined by unique single features emphasizes fine-grained control of outputs from the bottom-up dimensional modules, supposing that in the case of search for feature singletons, dimensional signals in the target dimension may be upweighted at the expense of those from irrelevant dimensions. One promising approach to accounting for the current data is to apply the dimensional-weighting processes at the heart of the dimension-weighting model to conjunction search, and to couple this with the idea of top-down, feature-based guidance that is at the core of the guided search model.

According to such an account of conjunction search, bottom-up computations of salience are constrained within dimension-specific modules. Then, following dimension weighting, signals from particular task-relevant dimensions may be up-weighted at the expense of other dimensions, explaining why there is no motion effect. Following guided search, top-down, feature-based control serves to prioritize locations containing relevant features. In addition to prioritizing locations containing relevant features, this feature-based guidance also serves to increase the influence of salient nontarget singletons. Applied to the case of orientation, the story runs as follows: When an orientation singleton is present, a bottom-up signal is present in the orientation map, and since orientation is task relevant, this is passed to the activation map. When this singleton is presented with the target colour, it receives top-down activation on the basis of its colour; in contrast, when it is presented in the nontarget colour, it does not receive top-down activation. Thus, an orientation singleton with target colour is both top-down and bottom-up salient, whereas one with nontarget colour is only bottom-up salient.

Colour singleton interference in the current experiment was weaker than orientation singleton interference and was not statistically significant when the colour singleton had a nontarget orientation. At first glance, the account detailed above would appear to have difficulty to account for this null effect of a colour singleton with the nontarget orientation. Surely, if top-down, feature-based control serves merely to increase activation of an already salient singleton, interference should be observed even when the singleton does not possess a target feature. The null result, however, is consistent with the feature-weighting account under two scenarios. Firstly, it might be that the colour singleton implemented here is a relatively weak source of interference in the context of a relatively salient conjunction target; as a result, the colour singleton does not reach the threshold required for interference, without top-down support. Secondly, it could be that feature-based weighting could take the form of *down-weighting* nontarget features in addition to *up-weighting* target features (e.g., Treisman & Sato, 1990; see Dent et al., 2012, for discussion of inhibitory coding in conjunction search). Thus, the salience of the singleton with the nontarget orientation could be actively reduced by top-down weighting processes.

The current results demonstrated interference from salient singleton distractors defined in *both* the orientation and colour dimensions as well as top-down modulation by *both* target orientation and target colour. Many previous authors have noted that colour provides a particularly compelling cue by which to organize a display in to subgroups; indeed, in their modelling work, Grossberg et al. (1994) assumed that colour–orientation search began with segmentation and grouping on the basis of colour. Likewise, Friedman-Hill and Wolfe (1995) showed that it is possible to use colour to efficiently find an odd orientation in a colour-defined subgroup.

The current results suggest that participants do not universally segment the display by one of the two features before looking for odd items in this subset. Had this been the case, if participants used colour as the primary cue before searching for orientation singletons, then we should have observed orientation interference that is modulated by colour, but no colour interference effect. If participants universally used orientation to select a subset of the display with target orientation before searching for colour singletons, then colour interference modulated by orientation should be observed without orientation interference. The fact that both types of interference were observed, and in each case the interference was modulated by the other feature, is more consistent with simultaneous use of top-down and bottom-up signals along both dimensions.

In order to address the possibility that some participants used colour to constrain orientation signals, whereas others used orientation to constrain colour signals, the correlations amongst different measures of performance are informative. If some participants used colour to group the display before examining bottom-up orientation contrasts, whereas other participants operated according to the opposite strategy, then there should be a negative correlation between singleton-distractor interference in the colour and orientation domains. However, considering both target-present trials and target-absent trials, there is no statistically significant correlation between interference across the three dimensions, and the direction of all correlations is positive.

A remaining possibility is that participants do indeed operate by initially segmenting the display by selecting items with the target feature along one or other of the dimensions (orientation or colour), but which dimension they use to drive this segmentation varies from trial to trial, according to specific stimulus characteristics and item layout. If participants did indeed operate in this fashion, no negative correlation between colour and orientation interference would be observed. However, there are good reasons to doubt that participants operate according to this strategy. Firstly, powerful intertrial priming effects have been documented in search including conjunction search, such that when the target is defined by the same features across trials performance is facilitated (e.g., Becker & Horstmann, 2009; Koshino, 2001; Kristjánsson et al., 2002). Such priming influences would seem to favour a consistent approach to segmentation rather than a variable one if that is what participants were doing. This switching strategy seems particularly unlikely given that experimentally induced switches in the dimension that defines the target in feature search are particularly disruptive to search (e.g., see Found & Müller, 1996). Secondly, neurophysiological data have demonstrated simultaneous additive enhancement of stimuli with target features in cases where multiple targets are to be selected (e.g., see Andersen et al., 2008, 2015). If simultaneous enhancement of target colour and orientation can permit efficient selection of groups of items, then it is difficult to explain why participants would not also use such a strategy in the case of selection of a single target. As a result, an account in terms of the simultaneous top-down weighting of multiple features is preferred over an account in terms of trial-by-trial switching, although further research will be needed to definitively rule out such an account.

Experiment 1 establishes that salient singleton distractors do in fact interfere with conjunction search, suggesting that there are important limits on top-down control of conjunction search. However, this single experiment does not allow us to really answer the question of the nature of this interference in relation to that observed in feature search (e.g., Kumada, 1999). Firstly, whilst the interference in conjunction search may be significant, it may be smaller than the interference observed in feature search (when closely matched displays are used). Secondly, the results of Experiment 1 clearly demonstrated that singleton interference was modulated by target features. However, it is unknown to what extent this modulation is specific to conjunction search or is in fact more general, applying also in the context of feature search. If these effects are specific to situations where distractors with target features compete against the target for selection, as participants use top-down signals to bias selection towards the target and away from distractors, they may be absent in feature search where the target is unambiguously signalled by a single feature. Such a pattern of results would underline the important contribution of top-down, feature-based control in conjunction search. In order to explore these issues, in Experiment 2 we explored singleton interference effects in a feature search task closely modelled on the conjunction search task of Experiment 1.

## Experiment 2: Feature search for a colour singleton

The purpose of Experiment 2 was to examine singleton-distractor interference in the context of a version of a feature search task with displays that were closely matched to those of Experiment 1. To this end, the displays of Experiment 1 were modified such that all the distractors with the exception of the target and the colour singleton distractor were the same colour. Thus, the target was defined explicitly as a colour singleton the only red (or green) item present in the display. Half of the distractors had one orientation (e.g., horizontal) whereas the target and the remaining distractors had another (e.g., vertical). What will be the magnitude of any interference effects in feature search, and will any interference be modulated by orientation?

### Method

#### Participants

Sixteen participants from the staff and students at the University of Essex volunteered in exchange for either course credit or for a payment of £5. There were seven male and nine female participants, all of whom were right-handed (mean age = 23.8 years, range: 18–33 years).

#### Equipment

The same equipment as Experiment 1 was used.

#### Stimuli, design, and procedure

Experiment 2 was identical to Experiment 1, except for the following exceptions. The displays of Experiment 1 were adapted to construct a colour feature search task (see Fig. 1 for an illustration of the displays used and Table 3 for an exhaustive list of the targets and distractors used). This was achieved simply by changing the colour of the distractors with the target colour to the nontarget colour. Thus, on singleton-distractor-absent trials, the target was a uniquely coloured item amongst items of a different uniform colour. Half the participants searched for a red target amongst green distractors, and half searched for a green target amongst red distractors. The orientation difference present in Experiment 1 was maintained, such that in the displays without singleton distractors, half the nonsingleton distractors and the target were vertical, whereas the other half of the nonsingleton distractors were horizontal. As in Experiment 1, three singleton distractors were used: colour singleton distractors (blue), orientation singleton distractors (45°), or motion singleton distractors (moving 1.8 cm/s). In the cases of the colour and motion singleton distractors, the singletons could appear either in the target orientation vertical or the nontarget orientation horizontal; orientation singletons did not vary in colour and were always presented in the colour of the nonsingleton distractors. All other aspects of the Experiment were as for Experiment 1.

**Table 3:** Search items and their features used in colour feature search (Experiment 2)

Group	Dimension	Target	Nonsingleton distractors		Singleton distractor		
			Target orientation	Nontarget orientation	Colour	Orientation	Motion
Group 1	Colour	Red	Green	Green	Blue	Green	Green
	Orientation	Vertical	Vertical	Horizontal	Vert / Horiz	45°	Vert / Horiz
	Motion	0 cm/s	0 cm/s	0 cm/s	0 cm/s	0 cm/s	1.8 cm/s
Group 2	Colour	Green	Red	Red	Blue	Red	Red
	Orientation	Vertical	Vertical	Horizontal	Vert / Horiz	45°	Vert / Horiz
	Motion	0 cm/s	0 cm/s	0 cm/s	0 cm/s	0 cm/s	1.8 cm/s

*Note.* Vertical and horizontal are occasionally abbreviated as *vert* and *horiz* due to space constraints

### Results

The data used in the analyses reported are publicly available online (https://osf.io/wbr2p/). Incorrect RTs (2.48%) and RTs > 2,000 ms (0.26%) were excluded (total 2.74%) from the RT analyses. Although accuracy was overall very high (see Table 4), for completeness, the same analyses are applied to both RT and accuracy data.

**Table 4:** Accuracy levels (proportion correct) in Experiment 2. A: data for trials where either a motion or colour singleton distractor was present. B: data for trials where either an orientation singleton distractor was present, or the singleton distractor was absent

A: Accuracy levels (proportion correct) in Experiment 2 showing colour and motion singleton-present trials								
	Target absent				Target present			
	Target orientation		Non-target orientation		Target orientation		Non-target orientation	
Colour	0.94	0.95	0.96	0.93	0.97	0.97	0.95	0.97
Motion	0.99	0.98	0.99	0.99	0.99	0.97	0.98	0.99
Display size	12	16	12	16	12	16	12	16
B: Accuracy levels (proportion correct) in Experiment 2 showing orientation singleton-present and singleton-absent trials								
	Target absent				Target present			
Orientation	1.00		0.98		0.98		0.97	
Singleton absent	0.98		0.98		0.98		0.97	
Display size	12		16		12		16	

#### Baseline search RTs

**Fig. 4:**
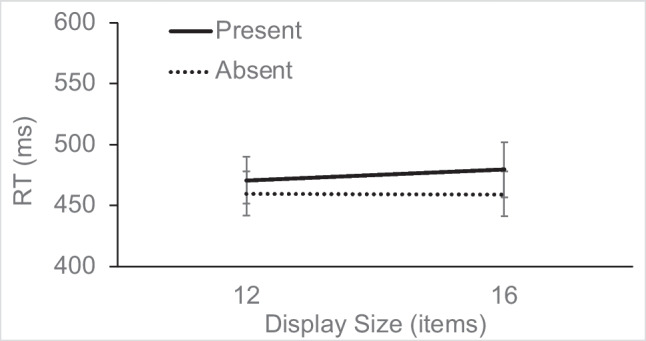
Experiment 2 search RTs for target-present and target-absent trials as a function of display size, on singleton absent trials (error bars show standard errors)

RT in the singleton-distractor-absent trials (see Fig. 4) was analyzed using a 2 × 2 repeated-measures ANOVA, with the factors target presence (present vs. absent) and display size (12 or 16). There were no statistically significant effects at all, target presence, *F*(1, 15) = 2.61, η_p_^2^ = 0.15, *p* = 0.127; display size, *F*(1, 15) = 1.35, η_p_^2^ = 0.08, *p* = 0.264; Target Presence × Display Size interaction, *F*(1, 15) = 2.53, η_p_^2^ = 0.14, *p* = 0.133. There was no increase in RT as a function of display size (search efficiency 1 ms/item overall), indicating parallel processing of the displays. It was also of interest compare overall baseline search RTs in the colour feature search of Experiment 2 with those observed in the conjunction search task of Experiment 1. A *t* test revealed that RTs were overall significantly faster in Experiment 2 than in Experiment 1, *t*(30) = 7.63, Cohen’s *d* = 2.70, *p* < 0.001.

Accuracy, an ANOVA with the same structure as that used to analyze RT, revealed no significant effects, *F*s(1, 15) < 3.94, η_p_^2^ < 0.21, *p* > 0.066.

#### Interference effects

**Fig. 5:**
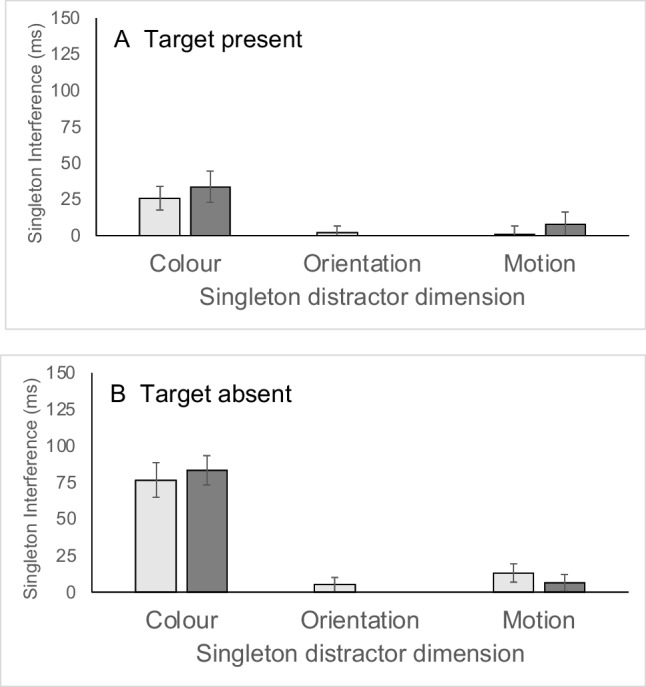
Experiment 2 singleton interference effects (singleton present − singleton absent) in milliseconds, as a function of singleton dimension (horizontal axis) and nonsingleton properties. *Note.* For colour and motion singletons, light-grey bars show interference scores for singletons that replaced distractors with the nontarget orientation, and dark-grey bars show singleton interference for singletons that replaced distractors with the target orientation. Orientation singletons by definition never shared the target orientation. Panel **A** shows data for target present trials, whereas Panel **B** shows data for target-absent trials

Singleton-distractor interference effects were calculated as the difference in RT or accuracy between singleton-distractor-present and singleton-distractor-absent responses (see Fig. 5 for RT). Interference effects for singletons in each of the three dimensions were analyzed separately using two or three factor ANOVAs, with the factors of target presence (target present vs. target absent), display size (12, 16), and nonsingleton features of the singleton distractor (target orientation, nontarget orientation) where relevant (e.g., in the motion and colour singleton cases).

##### Colour

Analyses of RT showed that the overall interference effect was greater than zero ms, *t*(15) = 7.05, Cohen’s *d* = 1.76, *p* < 0.001. An ANOVA with the factors target presence (target present vs. target absent), display size (12 or 16), and nonsingleton features of the singleton distractor (target orientation, nontarget orientation) revealed a main effect of target presence, *F*(1, 15) = 40.25, η_p_^2^ = 0.73, *p* < 0.001, the interference effect was larger (80 ms) for target-absent than for target-present (30 ms) trials, although both interference effects were significantly different from zero as indicated by one-sample *t* tests, *t*(15) = 9.08, Cohen’s *d* = 2.27, *p* < 0.001; *t*(15) = 3.45, Cohen’s *d* = 0.86, *p* = 0.004, for separate analyses of target-present and target-absent trials. No other main effect or interaction approached significance: nonsingleton features of the singleton target, *F*(1, 15) = 1.16, η_p_^2^ = 0.07, *p* = 0.298; display size, *F*(1, 15) = 0.00, η_p_^2^ = 0.00, *p* = 0.991; Target Presence × Nonsingleton Features of the Singleton Target, *F*(1,15) = 0.01, η_p_^2^ = 0.00, *p* = 0.944; Target Presence × Display Size, *F*(1, 15) = 1.56, η_p_^2^ = 0.09, *p* = 0.230; Nonsingleton Features of the Singleton Target × Display Size, *F*(1, 15) = 0.52, η_p_^2^ = 0.03, *p* = 0.481; Target Presence × Nonsingleton Features of the Singleton Target × Display Size, *F*(1,15) = 0.77, η_p_^2^ = 0.05, *p* = 0.409.

Since colour interference was significant in both Experiments 1 and 2, it was of interest to consider the relative magnitude of the interference across experiments. When the colour singleton-distractor interference effect on target-present trials in Experiment 2 was compared with that observed in Experiment 1, there was no statistically significant difference, *F*(1, 30) = 0.19, η_p_^2^ = 0.01, *p* = 0.66.

Analyses of accuracy showed that the overall interference effect was significant, *t*(15) = 3.00, Cohen’s *d* = 0.75, *p* = 0.009. An ANOVA with the same structure as that used to analyze RT revealed a main effect of target presence, *F*(1, 15) = 5.42, η_p_^2^ = 0.27, *p* = 0.034. The overall interference effect was significant for target-absent, *t*(15) = 2.94, Cohen’s *d* = 0.74, *p* = 0.010, but not for target-present trials, *t*(15) = 1.48, Cohen’s *d* = 0.37, *p* = 0.160. No other effects or interactions were significant, *F*s(1, 15) < 2.6, η_p_^2^ < 0.15, *p* > 0.131.

##### Orientation

Analyses of RT showed that the overall interference effect, was not significantly different from zero ms, *t*(15) = 1.14, Cohen’s *d* = 0.29, *p* = 0.272. An ANOVA with the factors target presence (target present vs. target absent) and display size (12 or 16) revealed no significant main effects or interactions: target presence, *F*(1, 15) = 0.19, η_p_^2^ = 0.01, *p* = 0.672; display size, *F*(1, 15) = 0.91, η_p_^2^ = 0.06, *p* = 0.355; target Presence × Display Size, *F*(1,15) = 0.17, η_p_^2^ = 0.01, *p* = 0.686.

Regarding accuracy, the overall interference effect was not significant, *t*(15) = 0.87, Cohen’s *d* = 0.22, *p* = 0.399. However, an ANOVA with the same structure as that applied to RT revealed an effect of target presence, *F*(1, 15) = 6.48, η_p_^2^ = 0.30, *p* = 0.022, but no other effects, *F*s(1, 15) < 2.60, η_p_^2^ < 0.13, *p* > 0.145. However, when tested separately, *t* tests revealed no significant interference effect for either target-absent, *t*(15) = 2.03, Cohen’s *d* = 0.51, *p* = 0.061, or target-present trials, *t*(15) = 0.25, Cohen’s *d* = 0.06, *p* = 0.805.

##### Motion

Analysis of RT showed that the overall interference effect was not significantly different from zero, *t*(15) = 1.70, Cohen’s *d* = 0.42, *p* = 0.110. An ANOVA with the factors target presence (target present vs. target absent), display size (12 or 16), and nonsingleton features of the singleton distractor (target orientation, nontarget orientation) revealed no significant main effects or interactions, target presence, *F*(1, 15) = 0.41, η_p_^2^ = 0.02, *p* = 0.534; nonsingleton features of the singleton distractor, *F*(1, 15) = 0.00, η_p_^2^ = 0.00, *p* = 0.995; display size, *F*(1, 15) = 0.06, η_p_^2^ = 0.01, *p* = 0.810; Target Presence × Features of the Singleton Distractor, *F*(1, 15) = 2.92, η_p_^2^ = 0.16,* p* = 0.108; Target Presence × Display Size, *F*(1,15) = 0.01, η_p_^2^ = 0.00, *p* = 0.923; features of the singleton distractor × display size, *F*(1,15) = 2.74, η_p_^2^ = 0.15, *p* = 0.119; Target Presence × Features of the Singleton Distractor × Display Size, *F*(1,15) = 0.17, η_p_^2^ = 0.01, *p* = 0.685.

Regarding accuracy, the overall effect of the presence of the singleton distractor was significant, *t*(15) = 3.30, Cohen’s *d* = 0.83, *p* = 0.005, but the nature of this effect was facilitatory, such that participants were very slightly more accurate when the singleton was present compared with when it was absent. An ANOVA with the same structure as that used to analyze RT revealed an interaction between nonsingleton properties of the singleton and display size, *F*(1, 15) = 7.94, η_p_^2^ = 0.35, *p* = 0.013. Separate analysis of data from the Display Size 12 trials showed that the overall effect of singleton-distractor presence was significant, *t*(15) = 2.34, Cohen’s *d* = 0.59, *p* = 0.033, but an ANOVA showed no effect of the nonsingleton properties, *F*(1, 15) = 1.15, η_p_^2^ = . 07, *p* = 0.300. At Display Size 16, whilst the overall effect of singleton-distractor presence was not significant, *t*(15) = 1.61, Cohen’s *d* = 0.4, *p* = 0.128, there was an effect of nonsingleton properties of the singleton distractor, *F*(1, 15) = 7.5, η_p_^2^ = 0.33, *p* = 0.015, such that the facilitation was greater than zero only when the singleton distractor did not share the target orientation, *t*(15) = 2.56, Cohen’s *d* = 0.64, *p* = 0.022, and not when it did share the target orientation, *t*(15) = 0.06, Cohen’s *d* = 0.02, *p* = 0.952. However, it is important to bear in mind that the magnitudes involved here are tiny (1.3% vs. 0%).

### Discussion

Participants detected the presence of a colour singleton target more quickly than the Orientation × Colour conjunctions of Experiment 1 (467 vs. 701 ms). This is consistent with feature search being a simpler task than conjunction search. The 234-ms delay may be the time required to implement top-down guidance processes in conjunction search. In keeping with previous studies (e.g., Chan & Hayward, 2009; Kumada, 1999), the pattern of results shows a strongly dimensional pattern. There were no interference effects at all caused by the presence of singleton distractors defined within the task-irrelevant dimensions of motion or orientation on RT, but singleton distractors defined within the task-relevant dimension of colour had a clear impact on RT performance. Motion distractor singletons had some small effect on accuracy, but this was not interference, but rather the presence of a motion distractor singleton caused some small amount of facilitation, both on target-present and target-absent trials, consistent with the motion singleton being easily and rapidly rejected during relatively early processing of the displays, although this small effect based on a high order interaction should be treated with caution. Interestingly, there was no tendency whatsoever for the interference effect caused by the presence of a colour singleton distractor to be reduced according to whether the singleton distractor shared the orientation of the target. Whereas Experiment 1 showed that the colour singleton-distractor-interference effect could be reduced from a significant 36 ms to a nonsignificant 5 ms according to whether it shared the target orientation or not, in the case of colour feature search, the effect remained undiminished at 30 ms for target-present trials regardless of orientation.

It is informative that in Experiment 2 there was no modulation of the colour-singleton-distractor interference effect by orientation. This contrasts with strong modulation in Experiment 1. This contrast demonstrates that the mere presence of two distinct orientations that may allow perceptual organization into two groups differing in orientation is insufficient to modulate singleton-distractor interference by itself. The most natural explanation of the difference between these two experiments is that top-down, feature-based guidance plays a much stronger role in Experiment 1. It is likely that, in the face of a high degree of competition from multiple distractors possessing target colour in conjunction search, it may be necessary to engage top-down control by orientation. In colour feature search, when no other distractor has target colour, orientation guidance is likely unnecessary and disabled.

It is of interest that the magnitude of the colour-singleton-distractor-interference effect on target-present trials in the feature search task of Experiment 2 was no larger than the magnitude of the equivalent effect in Experiment 1. This is consistent with an influence of bottom-up salience in the task-relevant dimension of a conjunction search that is as large as the influence in a faster and more efficient feature search. This is interesting, it is not only that singleton-distractor-interference effects in conjunction search are nonzero, contradicting Lamy and Tsal (1999), they are actually as large as equivalent (target present) effects in feature search. Thus, in this sense, the influence of top-down control, at least when viewed from the perspective of controlling interference from singleton distractors in task-relevant dimensions, is no greater in feature than in conjunction search.

## Experiment 3: Feature search for an orientation singleton

Experiment 2 investigated search for a colour feature, and whilst some colour singleton-distractor interference was observed there was no modulation of this by orientation. Experiment 1 generated much larger interference from orientation than colour singleton distractors. The goal of Experiment 3 was to examine orientation singleton-distractor interference in the context of orientation feature search. Will colour fail to modulate orientation singleton-distractor interference in the context of feature search or will colour modulation be observed in a context where singleton interference is greater, and participants may be motivated to control this interference.

### Method

#### Participants

Sixteen participants from the staff and students at the University of Essex volunteered in exchange for either course credit or for a payment of £5. There were five male and 11 female participants, one of whom was left-handed (mean age = 25.7 years, range: 19–35 years).

#### Equipment

The same equipment was used as for Experiments 1 and 2.

#### Design, stimuli, and procedure

The Experiment was identical to Experiment 2, except where noted below. The search displays were based on those used in Experiment 1 and adapted to form an orientation feature search (see Fig. 1 for an illustration of the displays and Table 5 for an exhaustive list of the targets and distractors used). In particular, all the distractor bars with the target orientation were changed to have the orientation of nonsingleton distractors. The colour difference in the display was maintained such that the distractors in the singleton-distractor-absent displays were either green or red. For one half of the participants, the target was a vertical red bar, and for the other half, it was a vertical green bar. Again, three salient singleton distractors were possible: a moving singleton, a coloured singleton (blue), and an orientation singleton (e.g., slanted 45 degrees). In the case of the orientation and the motion singleton distractors, colour varied with the distractor presented in the target colour half the time and the distractor colour half the time. In the case of colour the nonsingleton properties did not vary, and the singleton distractor was always blue and horizontal.

**Table 5:** Search items and their features used in orientation feature search (Experiment 3)

Group	Dimension	Target	Nonsingleton distractors		Singleton distractor		
			Target colour	Nontarget colour	Colour	Orientation	Motion
Group 1	Colour	Red	Red	Green	Blue	Red / Green	Red / Green
	Orientation	Vertical	Horizontal	Horizontal	Horizontal	45°	Horizontal
	Motion	0 cm/s	0 cm/s	0 cm/s	0 cm/s	0 cm/s	1.8 cm/s
Group 2	Colour	Green	Green	Red	Blue	Red / Green	Red / Green
	Orientation	Vertical	Horizontal	Horizontal	Horizontal	45°	Horizontal
	Motion	0 cm/s	0 cm/s	0 cm/s	0 cm/s	0 cm/s	1.8 cm/s

### Results

The data used in the analyses reported is publicly available online (https://osf.io/wbr2p/). All incorrect RTs (3.20%) and RTs > 2,000 ms (0.09%) were excluded (3.29% in total). Although accuracy was overall very high (see Table 6), for completeness, the same analyses are applied to both RT and accuracy data.

**Table 6:** Accuracy levels (proportion correct) in Experiment 3. A: data for trials with either an orientation or motion singleton distractor was present. B: data for trials where either a colour singleton distractor was present, or the distractor singleton was absent

A: Accuracy levels (proportion correct) in Experiment 3 showing orientation and motion singleton-present trials								
	Target absent				Target present			
	Target colour		Nontarget colour		Target colour		Nontarget colour	
Orientation	0.88	0.88	0.98	0.95	0.98	0.96	0.98	0.97
Motion	0.99	0.98	0.98	0.99	0.98	0.96	0.98	0.97
Display size	12	16	12	16	12	16	12	16
B: Accuracy levels (proportion correct) in Experiment 3 showing colour singleton-present and singleton-absent trials								
	Target absent				Target present			
Colour	0.99		0.98		0.96		0.96	
Singleton absent	0.98		0.98		0.97		0.96	
Display size	12		16		12		16	

#### Baseline search RT

**Fig. 6:**
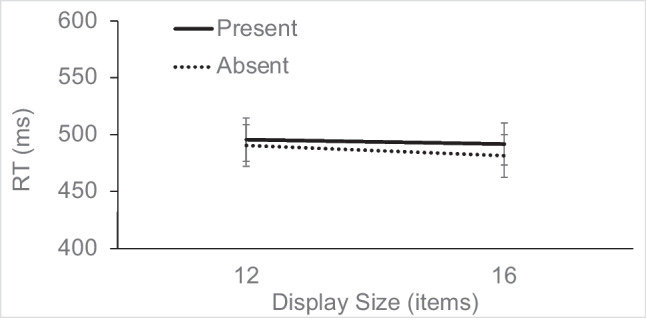
Experiment 3 search RTs for target-present and target-absent trials as a function of display size, on singleton absent trials (error bars show standard errors)

RT in the singleton-distractor-absent trials (see Fig. 6) was analyzed using a 2 × 2 repeated-measures ANOVA, with the factors target presence (present vs. absent) and display size (12 or 16). The main effect of display size was significant, *F*(1, 15) = 13.23, η_p_^2^ = 0.47, *p* = 0.002, indicating that RTs became significantly faster as display size increased. The slope of the function relating RT to display size was thus negative; however, the size of this slope was small at 2 ms per item (see Rangelov et al., 2017, for a thorough treatment of negative search slopes). The effect of target presence, *F*(1, 15) = 0.91, η_p_^2^ = 0.06, *p* = 0.354, and the Target Presence × Display Size interaction, *F*(1, 15) = 1.92, η_p_^2^ = 0.11, *p* = 0.186, were nonsignificant. It was also of interest to compare overall baseline search RTs in the orientation feature search of Experiment 3 with those observed in the conjunction search task of Experiment 1. A *t* test revealed that RTs were overall significantly faster in Experiment 3 than in Experiment 1, *t*(30) = 7.02, Cohen’s *d* = 2.48, *p* < 0.001.

Accuracy was analyzed using an ANOVA, with the same structure as that used to analyze RT. There was a main effect of target presence, *F*(1, 15) = 6.91, η_p_^2^ = 0.32, *p* = 0.019, and neither the effect of display size or the Display Size × Target Presence interaction were significant, *F*s(1, 15) < 2.90, η_p_^2^ < 0.15, *p*s > 0.100.

#### Interference effects

**Fig. 7:**
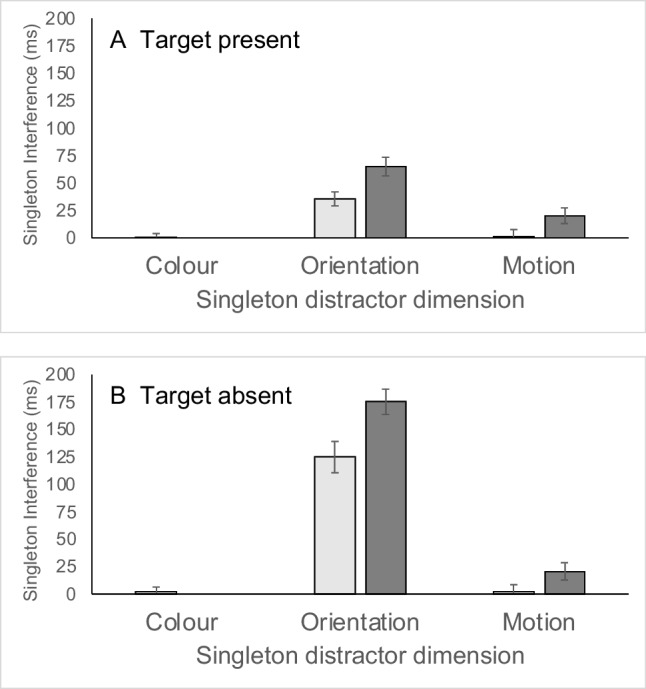
Experiment 3 singleton interference effects (singleton present − singleton absent) in milliseconds, as a function of singleton dimension (horizontal axis) and nonsingleton properties. *Note.* Light-grey bars show interference scores for singletons that replaced distractors with the nontarget colour, and dark-grey bars show singleton interference for singletons that replaced distractors with the target colour. Panel **A** shows data for target-present trials, whereas Panel **B** shows data for target-absent trials

Singleton-distractor-interference effects were calculated as the difference in RT or accuracy between singleton-distractor-present and singleton-distractor-absent responses (see Fig. 7, for RT). Interference effects for singleton distractors defined in each of the three dimensions were analyzed separately using two- or three-factor ANOVAs, with the factors of target presence (target present vs. target absent), display size (12, 16), and nonsingleton feature (target, nontarget), where relevant (e.g., orientation and motion).

##### Colour

The analysis of RT showed that the overall interference effect was not significantly different from zero, *t*(15) = 0.49, Cohen’s *d* = 0.12, *p* = 0.629. An ANOVA with the factors target presence (target present vs. target absent) and display size (12 or 16) revealed no significant main effects or interactions, target presence, *F*(1, 15) = 0.32, η_p_^2^ = 0.02, *p* = 0.578; display size, *F*(1, 15) = 0.26, η_p_^2^ = 0.02, *p* = 0.617; target Presence × Display Size, *F*(1, 15) = 0.00, η_p_^2^ = 0.00, *p* = 0.976.

Regarding accuracy, the overall interference effect was not significant, *t*(15) = 0.32, Cohen’s* d* = 0.08, *p* = 0.753. An ANOVA with the same structure as that used to analyze RT revealed no statistically significant effects, *F*s < 1.

##### Orientation

The overall interference effect was significantly different from zero, *t*(15) = 14.14, Cohen’s *d* = 3.54, *p* < 0.001. An ANOVA, with the factors target presence (target present vs. target absent), display size (12 or 16), and nonsingleton features of the singleton distractor (target colour, nontarget colour) revealed significant main effects of target presence, *F*(1, 15) = 93.04, η_p_^2^ = 0.86, *p* < 0.001, and nonsingleton properties, *F*(1, 15) = 16.79, η_p_^2^ = 0.53, *p* < 0.001. While the interference effect was larger when the target was absent than present (150 ms vs. 50 ms), the interference was significant regardless of target presence, *t*(15) = 13.96, Cohen’s *d* = 3.49, *p* < 0.001; *t*(15) = 8.15, Cohen’s *d* = 2.04, *p* < 0.001, for target-absent and target-present trials, respectively. Likewise, whilst the interference effect was larger when the singleton distractor appeared in the target colour than when it did not (120 ms vs. 81 ms), it was clearly significant regardless of colour, *t*(15) = 14.03, Cohen’s *d* = 3.51, *p* < 0.001; *t*(15) = 9.33, Cohen’s *d* = 2.33, *p* < 0.001, for target colour and nontarget coloured singleton distractors. There was also an interaction between target presence and display size, *F*(1, 15) = 4.81, η_p_^2^ = 0.24, *p* = 0.045. This appears to be driven by a minor tendency for interference to increase very slightly with increasing display size for target-present trials but to decrease with display size for target-absent trials. Since this minor interaction is tangential to the main concerns of this article, it will not be discussed further. No other effects or interactions were significant, display size, *F*(1, 15) = 0.13, η_p_^2^ = 0.01, *p* = 0.726; Target Presence × Nonsingleton Properties, *F*(1, 15) = 1.94, η_p_^2^ = 0.18, *p* = 0.115; Nonsingleton Properties × Display Size, *F*(1, 15) = 0.02, η_p_^2^ = 0.001, *p* = 0.886; three-way, *F*(1, 15) = 2.03, η_p_^2^ = 0.12, *p* = 0.175.

It is of interest to compare the extent to which colour modulates the orientation interference effect in Experiments 1 and 3. When experiment was added to the ANOVA described above as a between-participants factor, there was a significant three-way interaction between nonsingleton features of the singleton distractor, target presence, and experiment, *F*(1, 30) = 9.39, η_p_^2^ = 0.24, *p* = 0.005. Breaking the analysis down by target presence showed that, on target-present trials, the overall interference effect and the degree of modulation by colour was equivalent in both experiments, main effect of experiment, *F*(1, 30) = 1.37, η_p_^2^ = 0.04, *p* = 0.251, interaction between nonsingleton features of the singleton distractor and experiment, *F*(1, 30) = 2.46, η_p_^2^ = 0.08, *p* = 0.127. In contrast, on target-absent trials, whilst the overall interference effect was equivalent across experiments, main effect of experiment, *F*(1, 30) = 2.61, η_p_^2^ = 0.08, *p* = 0.116, there was greater modulation by colour in Experiment 1 than in Experiment 3, Experiment × Nonsingleton Features of the Singleton Distractor interaction, *F*(1, 30) = 33.75, η_p_^2^ = 0.53, *p* < 0.001.

Regarding accuracy, Table 3 indicates that there is a drop in accuracy when the orientation singleton appears in the target colour. The overall interference effect was significant, *t*(15) = 3.85, Cohen’s *d* = 0.96, *p* = 0.002. An ANOVA with the factors of target presence, display size, and nonsingleton feature (target colour, nontarget colour), revealed main effects of target presence, *F*(1, 15) = 14.00, η_p_^2^ = 0.483, *p* = 0.002, and nonsingleton feature, *F*(1, 15) = 10.67, η_p_^2^ = 0.42, *p* = 0.005, that interacted, *F*(1, 15) = 8.69, η_p_^2^ = 0.36, *p* = 0.010. Considering the target-present trials, the interference effect on accuracy was not significantly different from zero, *t*(15) = 1.35, Cohen’s *d* = 0.34, *p* = 0.197. In contrast, on target-absent trials, when the singleton was target coloured, there was a significant interference effect on accuracy, *t*(15) = 4.02, Cohen’s *d* = 1.01, *p* = 0.001, and when the singleton had the nontarget colour, the interference effect did not reach significance, *t*(15) = 2.06, Cohen’s *d* = 0.52, *p* = 0.057.

##### Motion

The overall interference effect was significant, *t*(15) = 2.53, Cohen’s *d* = 0.63, *p* = 0.023. An ANOVA with the factors target presence (target present vs. target absent), display size (12 or 16), and nonsingleton features of the singleton distractor (target colour, nontarget colour) revealed there was an effect of nonsingleton features of the singleton distractor, *F*(1, 15) = 9.12, η_p_^2^ = 0.38, *p* = 0.009. The singleton distractor created significant interference when it was target coloured, *t*(15) = 3.12, Cohen’s *d* = 0.78, *p* = 0.007, but created no interference when it had a nontarget colour, *t*(15) = 0.45, Cohen’s *d* = 0.11, *p* = 0.660. However, the magnitude of the interference is small at 21 ms. No other main effects or interactions were significant, target presence, *F*(1, 15) = 0.00, η_p_^2^ = 0.00, *p* = 0.967; display size, *F*(1, 15) = 0.22, η_p_^2^ = 0.01, *p* = 0.645; Target Presence × Nonsingleton Properties, *F*(1, 15) = 0.00, η_p_^2^ = 0.00, *p* = 0.999; Target Presence × Display Size, *F*(1, 15) = 0.03, η_p_^2^ = 0.002, *p* = 0.877; Nonsingleton Feature × Display Size, *F*(1, 15) = 0.38, η_p_^2^ = 0.03, *p* = 0.546; three-way, *F*(1, 15) = 0.39, η_p_^2^ = 0.03, *p* = 0.543.

Regarding accuracy, the overall interference effect was not significant, *t*(15) = 1.12, Cohen’s *d* = 0.28, *p* = 0.280, and there were no statistically significant effects in the ANOVA, *F*s < 1.

### Discussion

In Experiment 3, participants were able to detect the presence of an orientation singleton target more rapidly (490 ms) than they detected the presence of a colour × orientation singleton (701 ms). Again, suggesting feature search is an easier and perhaps less complex task than conjunction search. The overall RT for orientation search in Experiment 3 was similar to that observed for colour search in Experiment 2.

Again, the pattern of interference effects was strongly dimensional in nature. There was a very large interference effect caused by the presence of an orientation singleton distractor, whereas a colour singleton distractor had no significant effect. On the theoretically most important target-present trials, these interference effects were restricted to RT and did not appear for accuracy. Only on target-absent trials did an orientation singleton distractor presented in the target colour lead to a small increase in false alarms. Interestingly, in Experiment 3, the motion singleton distractor created a significant interference effect on RT. This finding is important since it serves to underline the fact that the motion singleton distractor used throughout all experiments reported here is in fact highly salient. That motion singleton distractors interfere in Experiment 3 with orientation singleton targets but not in Experiments 1 or 2 could suggest that relative to the salience of the colour singleton targets in Experiment 2, orientation singleton targets may be less salient, rendering them more susceptible to interference from the more salient motion singleton distractors. The results are generally in keeping with the findings in the literature, showing that shape defined targets to which orientation is related are generally more susceptible to dimension general interference than are colour defined targets. Alternatively, it could be that it is more difficult to control the dimensional weight on orientation independently of other dimensions, making it more difficult to use orientation to exclude salience from contrasts defined in the other dimensions.

In contrast to Experiment 2, participants also appear to make some use of colour information to control interference from other dimensions, such that both motion and orientation singleton-distractor interference can be modulated by target colour, even though target colour is incidental to the task, and the task can be solved without colour information. This difference may arise since it is generally more difficult to use orientation top-down to drive activation than it is to use colour. It is widely acknowledged that different features may have different effectiveness at constraining search, and that colour is generally more effective than orientation. A second reason may be that levels of interference are generally larger in Experiment 3 than they are in Experiment 2; orientation singleton distractors create a lot of interference, and motion singleton-distractors interfere too, thus, there is likely to be greater incentive to use colour to control interference. Thus, modulation of singleton interference by other features is not unique to conjunction search but can occur in search for a target defined by a single feature given the right features in the right context.

However, it is important to note that the extent to which colour modulates the orientation effect is much greater in Experiment 1 than it is in Experiment 3, at least on target-absent trials, where the interference effect in Experiment 1 changes from 33 to 208 ms, depending on singleton colour; in contrast, in Experiment 3, the change is from 125 to 176 ms.Thus, a key difference between the use of colour in Experiment 1 and Experiment 3 is that colour exerts a stronger modulatory influence in Experiment 1. In addition, far from the effect of a salient singleton distractor being exclusive to search for a target defined by a unique feature, the overall level of interference is statistically indistinguishable in colour feature search (Experiment 3) and conjunction search (Experiment 1).

Returning to the question of the mechanism by which top-down feature control may control bottom-up dimensional salience, the results of Experiment 3 are informative. The effect of orientation interference is indistinguishable in feature search and in conjunction search. This finding is clearly at odds with the idea of generally greater top-down, feature-based control in conjunction search leading to smaller interference effects compared with feature search. These issues are explored more thoroughly in the General Discussion.

## General discussion

Previous research revealed conflicting results regarding interference from task-irrelevant feature singletons in a conjunction search task. Whereas Lamy and Tsal (1999) found no significant interference, at least on target-present trials, Proulx (2007) found interference from a size singleton that was not modulated by the task relevance of its other features. Thus, the circumstances under which bottom-up salience plays a role in conjunction search remains unclear. In the study by Proulx (2007), the target and salient distractor-singleton coincided on a proportion of trials, and this may mean that participants are not fully motivated to ignore bottom-up salience signals. Whilst at first glance the results of Lamy and Tsal (1999) suggest that target selection in conjunction search is under complete top-down control by feature-based goals, under conditions in which the target and singleton do not coincide, close examination of this study leads to questions about whether participants were efficiently using top-down feature-based guidance, and whether the singletons used were adequately salient. This issue is important since if it were true that conjunction search is under complete top-down control (when explicit use of bottom-up signals is highly counterproductive), that would drive a wedge between feature and conjunction search, since feature search is often subject to interference from irrelevant singletons, especially when they are taken from a task-relevant dimension (e.g., Kumada, 1999; Liesefeld et al., 2019).

The current results challenge the conclusions of both Lamy and Tsal (1999) and Proulx (2007). The current results provide the first demonstration of robust singleton interference effects in conjunction search under conditions where the singleton is always a distractor. Thus, there are clear limits to the ability of participants to use top-down control to impede distractor interference in conjunction search. These interference effects, in contrast to the pattern of results reported by Proulx (2007), were strongly modulated by whether the singleton shared task-relevant features with the target, showing how top-down and bottom-up influences interact in conjunction search.

Importantly, across all three experiments, the pattern of interference was strongly dimensional in nature in line with the dimension weighting account (Liesefeld et al., 2019, 2021b). Thus, when the singleton distractor came from a task-relevant dimension, colour and orientation in the case of conjunction search and either colour or orientation in the feature search experiments, substantial interference was observed. This within-dimension interference was larger than any cross-dimension interference. It is thus clear that the signals that drive search in this context are not purely stimulus driven in the way envisaged in Theeuwes (2010) account of bottom-up processing in feature search, in which a dimension-independent global map of salience impervious to task goals drives early deployments of attention. Rather, the results suggest that when an irrelevant feature singleton is defined in a task-irrelevant dimension, its influence can be reduced often to zero (in the case of conjunction search or colour feature search). To give a concrete example, an irrelevant orientation singleton distractor creates significant interference in the case of conjunction search, but the same singleton distractor has no effect in the case of search for a colour singleton target. What is different across these tasks is the task relevance of the orientation dimension.

The results reported are thus largely in line with the core principles of the dimension weighting extension of the guided search model put forward by Müller and colleagues (e.g., Müller et al., 2003). Dimension weighting assumes that dimension-specific feature contrast signals are combined to produce a global salience or activation map. Dimension weighting is the process by which the weights assigned to the dimension-specific salience signals from each dimension may be flexibly increased or decreased according to task relevance or prior experience. In terms of the current results, increasing the dimensional weight applied to task-relevant dimensions substantially increases the interference caused by task-irrelevant feature singleton distractors defined in those dimensions. Indeed, Liesefeld et al. (2019) demonstrated that, in the context of a feature search for a target defined by a single feature, that interference from a singleton distractor defined in the same dimension as the target was much greater than that from a different dimension singleton distractor. The current results extend these dimensional effects to conjunction search and to feature searches with heterogeneous distractors.

The finding of RT disruption from a motion singleton distractor on search for orientation, even on target-present trials, makes the failure to find any such effect in the case of conjunction search or colour feature search more impressive, and serves to underline the substantial role for top-down control of bottom-up salience signals in these situations. Motion is clearly a salient signal in this context, but clearly also one that can be controlled in some circumstances. In order to account for the varying effects of motion across the feature search tasks, we suggest that the feature difference signal originating in the motion dimension is large compared with that originating in the orientation dimension (see Theeuwes, 1991, 1992, 2010) for a similar argument in the context of colour and shape); thus, when orientation is relevant and motion is task-irrelevant, the opportunity to observe interference is largest, and in this case even a top-down bias against motion is insufficient to prevent interference.

One characteristic of the current work is that the prevalence of the singleton distractor was relatively low since the distractor appeared only on 50% of trials, and within those 50% of trials there were three different possible singletons, giving a singleton prevalence of 16.66% for each singleton. Previous work (e.g., Müller et al., 2009; Zehetleitner et al., 2009) has shown how reducing distractor prevalence can lead to increased interference from a cross-dimensional distractor. However, it is important to acknowledge that this previous work used single-feature and not conjunction search tasks. No previous research has looked at the effects of distractor prevalence specifically in the case of conjunction search. The absence of cross-dimensional effects in conjunction search can then be explained by the particularly strong top-down weights that are applied in the colour and orientation dimensions. However, it must be acknowledged that our failure to measure cross-dimensional interference is essentially a null finding, and it remains possible that there were influences that we were unable to measure. Future research, using different or more sensitive methods, may uncover some subtle effects.

Regarding the single feature search tasks (Experiments 2 and 3), some cross-dimensional interference was observed in Experiment 3 from motion singletons, but no interference occurred in Experiment 2. The observation of motion interference in Experiment 3 (orientation search) but not Experiment 2 (colour search) is consistent with the prior literature, which suggests that colour targets are generally more salient than orientation targets (e.g., Theeuwes, 1992), and colour targets being more salient than orientation targets may then explain the absence of cross-dimensional effects in Experiment 2.

In neither Experiment 2 (colour target) nor Experiment 3 (orientation target) was cross-dimensional interference from orientation or colour singletons, respectively, observed. One reason that limited cross-dimensional interference was observed, despite relatively low distractor prevalence, is that our displays were composed of distractors that were heterogeneous in one of the nontarget dimensions (orientation in the case of a colour target, and colour in the case of an orientation target); such heterogeneity may well serve to reduce the saliency of the cross-dimensional distractor defined in the heterogenous dimension.

Aside from an absence of cross-dimensional interference in conjunction search, the main finding of Experiment 1 was that within-dimension interference along one dimension was strongly modulated by the target feature along the other dimension. Thus, colour singleton-distractor interference was observed *only* when the singleton appeared with the target orientation, and was absent when it had a nontarget orientation. Orientation singleton-distractor interference was present regardless of singleton-distractor colour, but it was 3 times larger on target-present and 6.5 times larger on target-absent trials when it shared target colour. These results suggest that information must be integrated across feature dimensions in order to explain how one dimension can constrain interference along another.

A natural account of this feature-based modulation of singleton interference can be given in the guided search framework. Within the guided search architecture, modulation could operate simply by adding top-down activation to bottom-up activation in a global activation map. The data from Experiment 1 would then be explained in the following way: When an orientation singleton is added to the display, it will have a high degree of bottom-up salience, larger than all the existing distractors. However, it will simultaneously lose all the top-down activation accruing from the orientation dimension since it does not have the target orientation. Changing the colour of the orientation singleton distractor between the target and nontarget colour will add top-down activation to this bottom-up activation. Thus, when the singleton has the target colour it will have both top-down and bottom-up activation, whereas all other distractors will have only top-down activation. The top-down plus bottom-up activation at the singleton distractor location will render it a powerful source of interference. The same story applies to the case of colour. In order to account for the null effect of motion the guided search notion of the summation of bottom-up and top-down signals must be supplemented with the idea of differential dimension weights being used to regulate the transmission of dimensional feature salience signals to the activation map (e.g., see Müller et al., 2003).

A very similar outcome would hold if instead of applying top-down activation universally across all dimensions, courtesy of a global activation map, top-down, feature-based information is able to differentially change the weight applied to a subset of the bottom-up signals originating in the dimensional modules, assumed by the dimension weighting account (e.g., Liesefeld & Müller, 2019; Müller et al., 2003). Indeed, Sauter et al. (2018) proposed a related extension to the dimension weighting account to explain changes in cross-dimensional interference as a function of distractor location probability. Essentially, such a mechanism constitutes a selective readout process where specific within-dimension bottom-up signals are given greater weight when they originate from locations highlighted by top-down signals, before being combined across dimensions. Interestingly, by situating a top-down influence at the location of the links between the dimensional modules and the activation map rather than at the level of the activation map, the selective gating process can apply to specific feature dimensions.

The current data do not really allow us to decide the issue of top-down modulation at the level of the global activation map (for all dimensions) or dimension-specific modulation for particular dimensions. However, the data from Experiment 3 are most relevant to this issue. In Experiment 3, modulation of interference from singleton distractors defined within the target-defining dimension was observed; however, modulation also spread to the singleton distractors defined within the motion dimension which did not define the target, and was completely task irrelevant in both feature and dimension. The most natural way to account for this pattern of data is to allow top-down modulation by colour of all locations rather than applying modulation only to the outputs of the target (orientation) defining dimension, perhaps by adding together top-down and bottom-up signals, as suggested in the guided search model.

It is also theoretically possible that top-down feature information is able to constrain computations in dimensional salience maps more directly (see Friedman-Hill & Wolfe, 1995, for discussion). On this account, orientation comparisons could be limited to operate within a colour-defined subset of locations, and colour comparisons could be limited to operate within orientation-defined subsets. If singleton interference had been much smaller in conjunction than in feature search, it would be difficult to argue for such second order parallelism in this task. It is tempting to take the very similar levels of interference observed in conjunction and feature search as evidence that both search tasks are fundamentally the same in terms of the bottom-up salience of the singleton in both cases. One version of this argument would be to suggest that in conjunction search orientation differencing is restricted to target coloured items, and colour differencing is restricted to target-oriented items. Such a scheme could operate if a top-down specified feature defined spatial template could be passed to and used to constrain processing of bottom-up dimensional contrast signals. However, given the current data, it is impossible to differentiate between an account where the underlying differencing operations are unchanged with some of these signals given an additive boost from top-down control, and an account where top-down control has a more fundamental effect on changing the processing within the dimensional modules.

The current results demonstrated interference effects in RT on both present and absent trials. However, there were some differences in the conjunction search data (Experiment 1) in terms of how interference from singleton distractors in different dimensions changed as a function of target presence. Thus, in the case of colour singleton distractors the interference did not increase on target-absent trials, whereas the interference from orientation singleton distractors did (so long as the singleton had the target colour). This pattern of findings may be explained by the relative activation of orientation and colour singleton distractors compared with the conjunction target. If the colour singleton distractor has a greater activation than the orientation singleton distractor, then it may suffer less competition from the presence of the target and show nonsignificant modulation by target presence.

### Summary

The current experiments demonstrated interference from salient singleton distractors defined in a task-relevant dimension in conjunction search, contradicting previous research that suggested null interference effects (e.g., Lamy & Tsal, 1999). The interference observed was as large as that observed in simpler feature search tasks. The results suggest that both feature and (efficient) conjunction search make use of dimension-specific salience signals that are computed bottom up. The results also suggest that these bottom-up salience signals are strongly modulated by top-down factors. Firstly, singleton interference was much greater from distractors defined in task-relevant dimensions than those defined in task-irrelevant dimensions. Secondly, top-down specification of target features modulated bottom-up interference, singletons with target features interfered more than those without. This feature-based modulation of dimension-specific salience played a more important role in conjunction than in feature search. The results can be accounted for within the guided search/dimension weighting framework. Dimension-specific salience signals are computed in dimensional modules, with the output from task-relevant modules weighted more highly than task-irrelevant modules. Target feature values also serve to highlight locations containing target features, and these signals also serve to emphasize the bottom-up salience signals originating at those locations. Whether that influence should be understood as addition of top-down and bottom-up feature maps, or more direct constraints on the fundamental feature computations, remains to be determined.

## Data Availability

All the data associated with the experiments reported here are available online (https://osf.io.wbr2p/).
